# Histone modifications rather than the novel regional centromeres of *Zymoseptoria tritici* distinguish core and accessory chromosomes

**DOI:** 10.1186/s13072-015-0033-5

**Published:** 2015-10-01

**Authors:** Klaas Schotanus, Jessica L. Soyer, Lanelle R. Connolly, Jonathan Grandaubert, Petra Happel, Kristina M. Smith, Michael Freitag, Eva H. Stukenbrock

**Affiliations:** Max Planck Institute for Terrestrial Microbiology, Karl-von-Frisch Strasse 10, 35043 Marburg, Germany; INRA, UMR 1290 INRA-AgroParisTech BIOGER, Avenue Lucien Brétignières, Thiverval-Grignon, 78850 France; Department of Biochemistry and Biophysics, Oregon State University, Corvallis, OR 97331-7303 USA; Christian-Albrechts University of Kiel, Environmental Genomics, Am Botanischen Garten 9-11, 24118 Kiel, Germany; Max Planck Institute for Evolutionary Biology, August-Thienemann-Str. 2, 24306 Plön, Germany

**Keywords:** Centromere, Histone methylation, ChIP-seq, Accessory chromosomes, *Zymoseptoria tritici* (*Mycosphaerella graminicola*)

## Abstract

**Background:**

Supernumerary chromosomes have been found in many organisms. In fungi, these “accessory” or “dispensable” chromosomes are present at different frequencies in populations and are usually characterized by higher repetitive DNA content and lower gene density when compared to the core chromosomes. In the reference strain of the wheat pathogen, *Zymoseptoria tritici*, eight discrete accessory chromosomes have been found. So far, no functional role has been assigned to these chromosomes; however, they have existed as separate entities in the karyotypes of *Zymoseptoria* species over evolutionary time. In this study, we addressed what—if anything—distinguishes the chromatin of accessory chromosomes from core chromosomes. We used chromatin immunoprecipitation combined with high-throughput sequencing (“ChIP-seq”) of DNA associated with the centromere-specific histone H3, CENP-A (CenH3), to identify centromeric DNA, and ChIP-seq with antibodies against dimethylated H3K4, trimethylated H3K9 and trimethylated H3K27 to determine the relative distribution and proportion of euchromatin, obligate and facultative heterochromatin, respectively.

**Results:**

Centromeres of the eight accessory chromosomes have the same sequence composition and structure as centromeres of the 13 core chromosomes and they are of similar length. Unlike those of most other fungi, *Z. tritici* centromeres are not composed entirely of repetitive DNA; some centromeres contain only unique DNA sequences, and *bona fide* expressed genes are located in regions enriched with CenH3. By fluorescence microscopy, we showed that centromeres of *Z. tritici* do not cluster into a single chromocenter during interphase. We found dramatically higher enrichment of H3K9me3 and H3K27me3 on the accessory chromosomes, consistent with the twofold higher proportion of repetitive DNA and poorly transcribed genes. In contrast, no single histone modification tested here correlated with the distribution of centromeric nucleosomes.

**Conclusions:**

All centromeres are similar in length and composed of a mixture of unique and repeat DNA, and most contain actively transcribed genes. Centromeres, subtelomeric regions or telomere repeat length cannot account for the differences in transfer fidelity between core and accessory chromosomes, but accessory chromosomes are greatly enriched in nucleosomes with H3K27 trimethylation. Genes on accessory chromosomes appear to be silenced by trimethylation of H3K9 and H3K27.

**Electronic supplementary material:**

The online version of this article (doi:10.1186/s13072-015-0033-5) contains supplementary material, which is available to authorized users.

## Background

“Accessory chromosomes” are considered not essential for the survival and reproduction of an organism [1]. Such “B” chromosomes have been described in many different organisms representing all major groups of eukaryotes [2], and are called “conditionally dispensable”, “lineage-specific”, or “accessory” chromosomes in fungi [3–6]. One unifying characteristic of accessory chromosomes in fungi is that they can be present or absent in different individuals in a given population and thus occur at variable frequencies [4]. In most cases, the functional importance of accessory chromosomes under natural conditions is unknown; however, the fact that they are maintained in some populations over evolutionary times suggests that they convey functional relevance, at least occasionally [7]. Accessory chromosomes that result in adaptive advantage have been characterized in several fungi and special attention has been drawn to the presence of pathogenicity determinants, for example in *Fusarium solani* MPVI (*Nectria haematococca*) [3, 5], *Fusarium oxysporum* f. sp. *lycopersici* [6] and *Leptosphaeria maculans* [8, 9]. In all species studied so far, accessory chromosomes are distinguished from core chromosomes by their high repeat content and low gene density as shown by chromosome staining and biochemical methods [2, 10]. Currently, no studies on the chromatin structure of accessory chromosomes in fungi are available.

In the reference strain of the wheat pathogen *Zymoseptoria tritici* (synonym *Mycosphaerella graminicola*), eight accessory chromosomes have been found, ranging from ~0.4 to 1 Mb in size [11]. Although these chromosomes comprise as much as 12 % of the total genome and encode more than 700 genes, no functional relevance has been assigned to accessory chromosomes in this species [11, 12]. This is consistent with recent analyses of transcriptomes and proteomes that revealed a majority of these genes to be non-transcribed (“silent”) during in vitro growth on rich medium, as well as during colonization of the wheat host [13, 14]. Accessory chromosomes may provide a special type of “genome niche”, conducive for rapid adaptive evolution of virulence-related genes, perhaps due to fewer selective constraints on sequence evolution [15]. Indeed, genes located on the *Z. tritici* accessory chromosomes appear to evolve considerably faster compared to genes on the core chromosomes [16]. The accessory chromosomes of *Z. tritici* were hypothesized to consist of duplicated sequences from core chromosomes that degenerated over time [11]. Our detailed analyses of chromosome content to infer the extent of paralogy on core and accessory chromosomes showed that the majority of genes on accessory chromosomes are unique [12, 14]. Thus, evolution and dynamics of these chromosomes in *Z. tritici* are apparently not driven by simple duplications or translocations from the core chromosomes, but rather caused by a complex interplay of frequent structural rearrangements, likely aided by the activity of repetitive elements [14, 17]. Closely related species of *Zymoseptoria* also contain accessory chromosomes that are at least partially homologous to those of *Z. tritici* [18]. This supports ancestral origins of *Zymoseptoria* accessory chromosomes and suggests that they, as the core chromosomes, have been maintained in populations of *Z. tritici* during speciation of the pathogen on wheat.

Accessory chromosomes are less faithfully transmitted than core chromosomes [5, 6, 11, 17, 19]. Transmission, and thus stability of eukaryotic chromosomes, derives in large part from two specialized regions, centromeres and telomeres (reviewed in [20]). Telomeric DNA repeats of *Z. tritici* are identical to the most common human repeat, 5′-(TTAGGG)_n_-3′ and have been found at 41 termini of the 21 chromosomes in the reference isolate IPO323 [11]. Few complete fungal centromeres have been identified [21], as centromeres are not defined by specific DNA sequence, even in the short, one nucleosome-long point centromeres of *Saccharomyces* and related species [22]. Most centromeres, however, are functionally defined by presence of the centromere-specific histone H3, CENP-A [23–25], which is called CenH3 in fungi [21]. Regional centromeres extend over kilo- to megabases and show large variation in sequence composition and chromatin organization [21, 22, 26]. The two known extremes in the filamentous fungi are *Neurospora crassa* centromeres, which are large (150–300 kb), AT-rich and enriched with trimethylated H3 lysine 9 (H3K9me3), a histone mark typically associated with gene silencing [27], and *Candida albicans* centromeres that are short (4–18 kb) and do not contain conserved motifs or AT-rich repeats [28].

To understand what causes the frequent meiotic or mitotic instability of *Z. tritici* accessory chromosomes, we analyzed centromeres, telomeres and subtelomeric regions. We used chromatin immunoprecipitation (ChIP) of CenH3 tagged with GFP in combination with high-throughput sequencing (ChIP-seq) to identify the centromeres of core and accessory chromosomes. We also asked if centromeres in *Z. tritici* were associated with transcriptionally active (euchromatic) or silent (heterochromatic) regions and tested for the presence of telltale histone modifications by ChIP-seq. Here, we show that centromeres and subtelomeric regions of accessory chromosomes and core chromosomes are similar, but that the overall distribution of euchromatic and heterochromatic histone modifications is significantly different between the two types of chromosomes.

## Results

### CenH3 is localized at several chromocenters in interphase nuclei

**Fig. 1:**
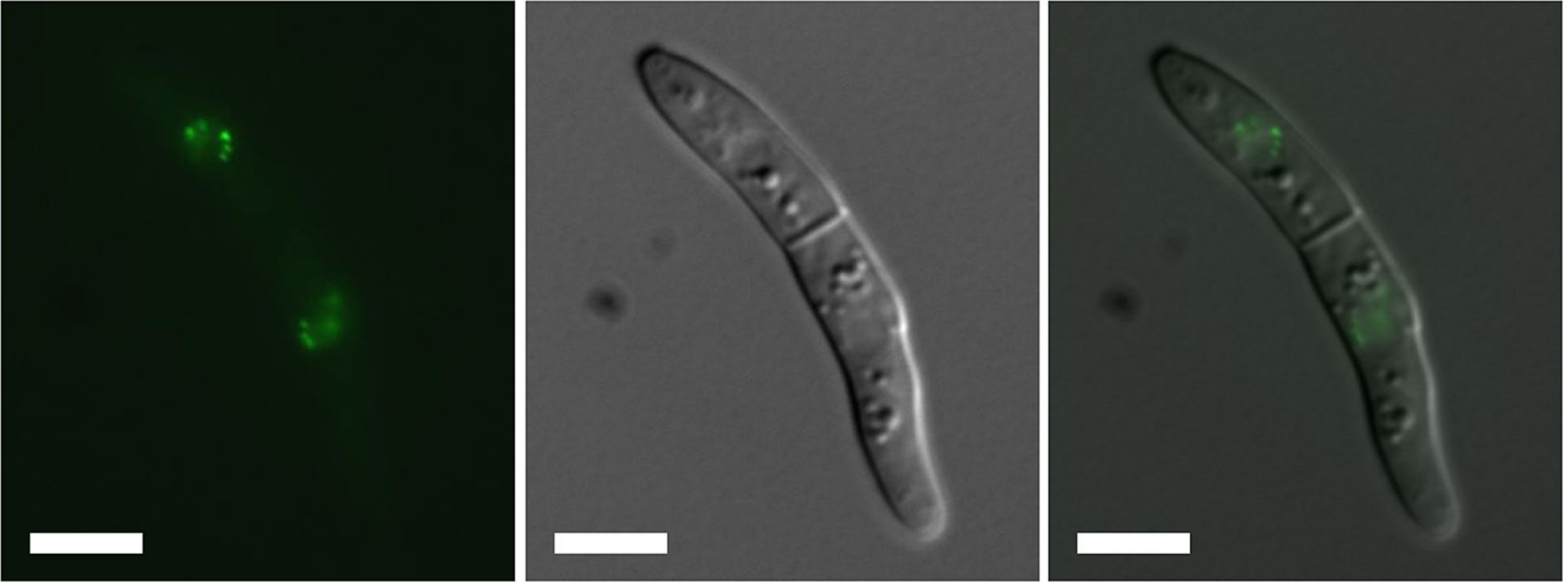
CenH3 is located in distinct foci in the nucleus. Strain Zt121-85 was grown on YMS medium for 24 h at 18 °C; images from a representative spore are shown. CenH3 is localized inside the nucleus and organized in several spots (between 4 and 7 foci can be distinguished by epifluorescence microscopy); there were no spores with single chromocenters. *Left,* Fluorescence microscopy of GFP–CenH3. *Center,* Bright field image. *Right,* Merged images. *Scale bar* 5 μm

To identify the nuclear localization of centromeres in *Z*. *tritici* nuclei, we tagged the centromere-specific histone CENP-A/CenH3 [24, 25]. We searched for a gene encoding CenH3 in the genome of the reference *Z. tritici* isolate IPO323 by BLASTP analyses with the *N. crassa* CenH3 homolog (NCU00145) [27] as query sequence. We identified Mgr74593 [11], now called Zt09_chr8_00234 [12], as a putative homolog. For *Agrobacterium tumefaciens*-mediated transformation, we used IPO323∆KU70 (Zt84), an IPO323 derivative in which the *KU70* gene had been deleted to eliminate non-homologous end joining (NHEJ) [29] and our IPO323ΔChr18 (Zt9) strain, in which chromosome 18 has been lost [14]. Strains without KU70 homologs yield correctly targeted transformants more easily than wild-type strains, but they also carry the risk of unwanted chromosome or chromatin aberrations, thus we used both *KU70*^+^ and *KU70*^−^ strains as transformation hosts. We generated strains with an N-terminal GFP fusion of CenH3 (Additional file 1: Figure S1); C-terminal CenH3–GFP fusions were unsuccessful in our hands (data not shown). The GFP-tagged allele was inserted at the endogenous CenH3 locus and is under the control of the native promoter. Transformed strains were made homokaryotic by repeated single spore isolations. We confirmed correct integration of the GFP construct by an initial PCR screen followed by Southern analyses and appropriate translation of the GFP-tagged CenH3 protein by western blot analyses (Additional file 1: Figure S1). To visualize and localize the centromeres of *Z. tritici*, we used fluorescence microscopy. We observed multiple discrete GFP–CenH3 signals concentrated in four to seven foci per interphase nucleus. This suggests that *Z. tritici* centromeres occur in several stable chromocenters rather than one single chromocenter (Fig. 1). We compared the average number of foci between GFP–CenH3 strains with a full chromosome complement (Zt118) and those that lack chromosome 18 (Zt121). Preliminary results suggest that the median number of foci observed, 6.56 and 6.84, respectively, is not statistically different between Zt118 and Zt121 strains (Wilcox sum rank test, *p* < 0.09; 708 and 684 foci counted, respectively). Instead, we saw minor changes in the number of bright foci.

### Centromeres of *Z. tritici* core and accessory chromosomes

**Fig. 2:**
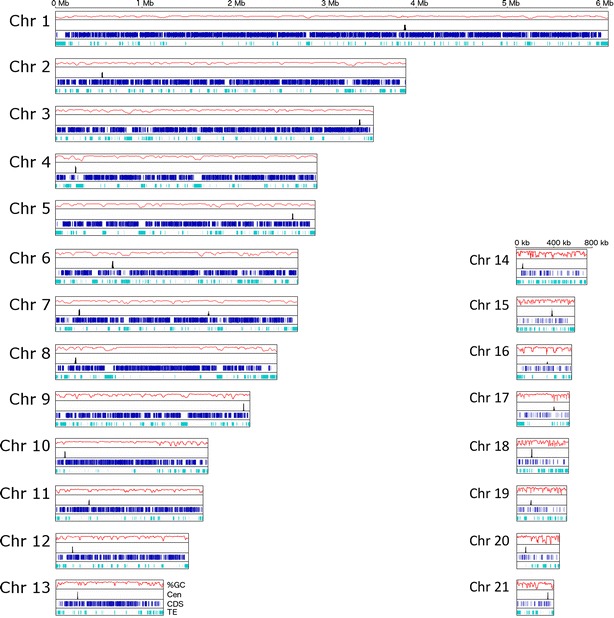
Centromeres are not located in the longest AT-rich region. All 21 chromosomes are drawn to scale and the ruler indicates the length of the chromosomes (Core chromosomes in Mb, accessory chromosomes in kb). For each chromosome, the GC-content (%GC, *red*), centromere position as determined by GFP–CenH3 enrichment (Cen, *black*), coding sequences (CDS, *blue*) and active or inactive transposable elements (TE, *marine*) are shown. Centromeres in *Z. tritici* are small, ranging from 5.57 kb (Cen13) to 13.55 kb (Cen8). Regions with low GC % are enriched with TEs. Centromeres are not located in the longest AT-rich regions for both core (Chr 1-13) and accessory (Chr 14-21) chromosomes. The apparent second, smaller peak on Chr 7 coincides with two rDNA repeats (positions 1,676,706-1,682,207 and 1,684,968–1,690,469, interrupted by a 2.76 kb intergenic spacer). Of ~50 actual repeats only two are included in the current genome assembly, thus identical reads stack at these positions to yield a false CenH3 peak (for details see also Fig. 6)

**Fig. 3:**
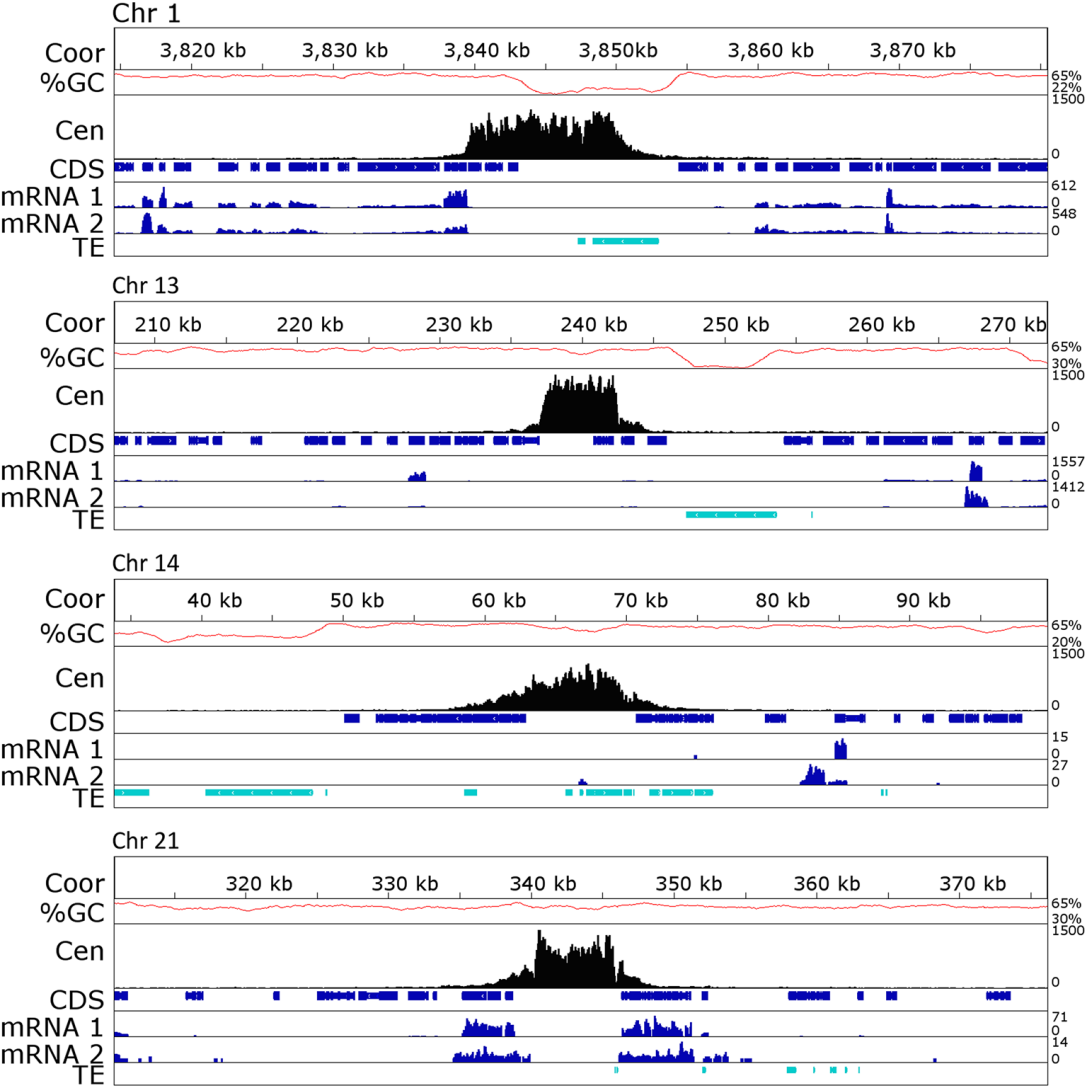
Centromeres of *Z. tritici* are small and contain expressed genes. Segments (65 kb) of core chromosomes (Chr) 1 and 13 and the accessory Chr 14 and 21 are shown. The* ruler* indicates chromosome coordinates (Coor, in kb), and GC-content (%GC, *red*), centromere position determined by GFP–CenH3 enrichment (Cen, *black*), location of coding sequences (CDS, *blue*) and active or inactive transposable elements (TE, *marine*) are shown. Expression data [14] from pure culture (mRNA1) and *in planta* at 4 days post-infection (mRNA2) are also plotted (*blue*). Genes surrounding or overlapping the centromere are expressed to varying degrees. AT-richness and enrichment with TEs is not a characteristic of *Z. tritici* centromeres

We used anti-GFP antibody in ChIP experiments with GFP–CenH3 strains to identify the centromeric DNA of core and accessory chromosomes. The DNA associated with CenH3 was sequenced on an Illumina HiSeq 2000 sequencer (Additional file 2: Table S1). Reads were processed, filtered and mapped to the IPO323 reference genome [11] as described in the Materials and Methods. Mapping of GFP–CenH3 reads revealed a single strong peak per chromosome, clearly showing the position of each centromere (Figs. 2, 3, Additional file 3: Figure S2, Additional file 4: Figure S3). To validate that the sequences were correctly mapped to the reference genome, we confirmed the lengths and positions of all centromeres by PCR analyses with centromere-specific primers designed to amplify fragments inside and outside of the centromeres (Additional file 5: Table S2).

There was no difference in centromere position for any of the chromosomes when we compared transformants derived from IPO323ΔChr18 (Zt9) and IPO323∆KU70 (Zt84); as expected, no reads mapped to accessory chromosome 18 of IPO323ΔChr18. Most of the core chromosomes of *Z. tritici* are acrocentric or near-acrocentric, whereas accessory chromosomes are mostly metacentric (Table 1, Additional file 6: Figure S4). We defined “metacentric” as chromosomes with centromeres that lie in the middle third, i.e., between 33 and 66 % of the total chromosome length. By this definition, chromosomes 1, 15, and 16 are metacentric, chromosomes 6, 11, 13, 17, 18, 19, and 20 are near-metacentric (between 20 and 33 % of the distance from the respective chromosome end), chromosomes 2, 12, and 21 are near-acrocentric (between 10 and 20 % of the distance from the respective chromosome), and chromosomes 3, 4, 5, 7, 8, 9, 10, and 14 are acrocentric (between the end and 10 % of chromosome length) (Additional file 6: Figure S4).

**Table 1:** Centromeres of *Z. tritici*

Chr.	Chr. length (bp)	Cen start (bp)	Cen stop (bp)	Cen length (bp)	Cen (% chr.)	AT (%)		Repeats in Cen (%)	Cen location (% chr.)
						Cen	Chr.		
1	6,088,797	3,839,299	3,851,749	12,450	0.20	56.3	46.9	28.2	37.0
2	3,860,111	5,12,901	5,21,916	9015	0.23	54.1	47.6	32.4	13.3
3	3,505,381	3,348,307	3,356,535	8228	0.23	50.4	47.4	0.0	4.4
4	2,880,011	2,17,113	2,26,545	9432	0.33	49.8	47.8	9.9	7.5
5	2,861,803	2,604,117	2,614,736	10,619	0.37	58.0	48.0	29.2	2.4
6	2,674,951	6,25,186	6,37,601	12,415	0.46	53.6	48.6	40.6	23.3
7	2,665,280	2,55,824	2,66,207	10,383	0.39	48.8	47.2	0.0	9.6
8	2,443,572	2,13,892	2,27,444	13,552	0.55	53.3	48.3	36.7	8.8
9	2,142,475	2,067,589	2,076,063	8474	0.40	53.6	48.5	0.5	3.0
10	1,682,575	99,716	1,09,365	9649	0.57	51.5	47.5	15.3	5.9
11	1,624,292	3,65,130	3,73,557	8427	0.52	59.8	47.2	44.2	22.5
12	1,462,624	1,80,233	1,88,209	7976	0.55	48.6	47.7	3.0	12.3
13	1,185,774	2,36,993	2,42,558	5565	0.47	47.3	48.0	0.5	19.9
14	7,73,098	59,960	70,870	10,910	1.41	46.8	51.5	28.5	7.8
15	6,39,501	3,82,500	3,94,754	12,254	1.92	50.3	49.0	18.9	40.2
16	6,07,044	3,32,004	3,42,592	10,588	1.74	57.2	48.5	36.0	37.3
17	5,84,099	4,06,958	4,18,893	11,935	2.04	57.6	48.0	26.1	29.8
18	5,73,698	1,59,000	1,71,999	12,999	2.27	51.9	50.8	3.2	27.9
19	5,49,847	1,48,227	1,59,387	11,160	2.03	54.5	48.7	0.8	27.0
20	4,72,105	94,677	1,05,169	10,492	2.22	45.9	48.5	9.5	20.1
21	4,09,213	3,40,264	3,46,657	6393	1.56	47.7	48.1	1.9	15.4

Chromosome (Chr.) length, centromere (Cen) position (Cen Start and Cen Stop), Cen length are shown. The relative sizes of centromeres as fraction of chromosome lengths (Cen  % chr.) were determined by ChIP-seq analyses. Chromosomes 1 to 13 are considered core chromosomes, and Chr. 14 to 21 are considered accessory chromosomes. Note that the average size of centromeres is not different between core and accessory chromosomes. The centromeric and chromosomal AT and repeat content is shown. Note that the overall AT-content and that of the centromeric DNA between core and accessory chromosomes does not vary and that there are overall few repeats in *Z. tritici* centromeres. Centromeres of core chromosomes are closer to chromosome ends than those of accessory chromosomes (expressed as the percentage distance from the chromosome mid-point; Cen location as % of chromosome length)

### Centromeres are not located in the longest AT-rich regions

Centromeres of *Z. tritici* are extremely short for a filamentous fungus, on average only 10.3 kb (Fig. 2; Table 1), but ranging from 5.57 kb (Chr. 13) to 13.55 kb (Chr. 8). The length of centromeric DNA is independent of chromosome length, which varies from 6 to 0.41 Mb. Compared to the very AT-rich centromeres of most other eukaryotes that have been studied, the GC-content of centromeres is little lower than the genome average, 48.3 % compared to 52.3 % (Figs. 2, 3, Additional file 3: Figure S2, Additional file 4: Figure S3; Table 1). In contrast to the long centromeres of *N. crassa* [27] and several fusaria [7, 21, 30], the centromeres of *Z. tritici* are also not located in the longest AT-rich region on each chromosome (Figs. 2, 3, Additional file 3: Figure S2; Additional file 7: Table S3). In total, we identified 847 AT-rich regions with an AT percentage above 50 %, covering 7.38 Mb (or 18.6 %) of the whole genome (Additional file 7: Table S3). These regions contain predominantly repetitive elements, but few coding sequences. They range in length from 2 kb to 86.3 kb with an average length of 9.7 kb, and an average AT-content of 55 %. Compared to the 11.6 % of DNA contained in accessory chromosomes, they harbor 21 % of the total AT-rich DNA and 23 % (196/847) of AT-rich regions. The longest AT-rich region per chromosome varies from 14.2 kb for chromosome 17 to 86.3 kb for chromosome 4. Only centromeres 1 and 5 are almost completely located in an AT-enriched region, but 17 other centromeres have AT-rich regions inside the CenH3-binding region.

### The centromeres of *Z. tritici* are not defined by a consensus motif

To determine if there are conserved motifs within centromeric DNA of *Z. tritici*, we used BLAST [31] and MEME [32] analyses to search for conserved sequences and motifs. We first masked repetitive sequences in the centromeres and included only non-repetitive DNA in the BLAST analyses. Both the blast analysis and the MEME motif search failed to identify any conserved centromere-specific motif in the *Z. tritici* genome. Repetitive DNA found in some centromeres belongs to various repeat families—no single category is present at all centromeres. None of the retrotransposons, DNA transposons or their relics are specific for centromeres, i.e., they occur also in chromosome arms.

### Centromeres of *Z. tritici* are not enriched in transposable elements (TEs)

The long AT-rich regions found in many fungi are composed of retrotransposons or relics of retrotransposons, and centromeres described in other filamentous fungi are composed almost entirely of repeats [21, 27, 30, 33]. In contrast, centromeres of *Z. tritici* are not composed of repetitive DNA as the mean repeat content of centromeric DNA is only 17.4 %, and centromeres 3, 7, 9, 13 and 19 have a repeat content of <1 % (Additional file 8: Table S4). Cen11 is the most repeat-rich centromere with a repeat content of only 44.2 %. Centromeres 3, 7, 9, 13 and 19 completely lack transposable elements (TEs) or relics of TEs, while even centromeres 2, 6, 8, 11 and 16 have TE coverage of only ~30 % (Additional file 8: Table S4). Repeat regions are overall short and can be grouped into a total of 17 different TE-families (Additional file 8: Table S4; [34]). RYN1 and unclassified repetitive regions (called “NoCat”) cover the most centromeric sequence on core chromosomes (each ~6 %), while—mostly inactive—RLG8 and RLC9 retroelements and DNA transposons are found most often at centromeres of accessory chromosomes. Overall, however, our comparisons of sequence motifs, AT-content, repeat content, and length revealed no significant differences between centromeres of core and accessory chromosomes.

### Centromeres of *Z. tritici* contain predicted and expressed genes

A total of 39 putative genes lie completely within or overlap centromeric DNA, as determined by the presence of previously predicted reading frames [11, 14]. We re-analyzed existing data [14] and mapped them to our new annotation [12]. Based on this analysis, 26 centromeric genes were transcribed (at reads per kilobase per megabase [RPKM] ≥2) during axenic growth in rich medium suggesting some functional relevance of these genes for the fungus (Additional file 9: Table S5). However, the mean RPKM value of centromeric genes on the core (385.4 ± 18881.5), and especially the accessory (1.7 ± 2.8) chromosomes is much lower than values obtained for all genes on core (584.9 ± 1992.5) or accessory chromosomes (23.0 ± 90.1); the mean RPKM for all genes on all chromosomes is 550.4 ± 1935.1 (Additional file 9: Table S5). Two genes completely within the centromeric DNA and three genes overlapping centromeric DNA, all on core chromosomes, showed consistent expression comparable to weakly expressed genes on chromosome arms. Only two have similarities to known genes, one encoding an alcohol dehydrogenase, the other an aminobutyrate aminotransferase; the remaining three are predicted or hypothetical proteins (Additional file 9: Table S5).

### Telomere repeat tracts of core and accessory chromosomes are similar

Because we found no significant differences between centromeres of core and accessory chromosomes, we next turned our attention to chromosome ends. Telomeres are considered refractory to standard cloning procedures, yet we found telomere repeats (TTAGGG_n_) in the published *Z. tritici* genome sequence to be 128 ± 20.1 bp long, thus slightly longer than the 120 bp previously found in *M. oryzae* [35] and *N. crassa* [36], or the 110 bp in *A. nidulans* [37] by more sophisticated methods. Telomeres on core and accessory chromosomes are comprised of similar numbers of repeats (22.3 ± 3.2 vs. 19.8 ± 3.3 TTAGGGs, respectively) and are thus of similar average lengths (134 ± 18.8 bp vs. 118 ± 19.7 bp, respectively; Additional file 10: Table S6). The total telomere tract length varied from 91 to 175 bp on core and 73 to 143 bp on accessory chromosomes. We noticed the presence of a near-standard repeat, TGAGGG, on 13 of 41 cloned ends, and in some cases there were tandem repeats of 10–14 copies present, which were slightly enriched on accessory chromosomes. We also found 51 interstitial TTAGGG repeats with a minimum length of three repeat units; 29 were on core, 22 on accessory chromosomes, while two core chromosomes (Chr. 3 and 8) had no such repeats (data not shown). Only five of these tracts had six or more repeats, and four are located within subtelomeric regions. In the absence of more detailed studies on telomere length from a larger collection of wild-type strains, we deem the small differences in average telomere tract length not significant, even though the difference in repeat length is similar to the decrease in repeat length observed between *Aspergillus* wild-type and *ku70* mutants [37]. We conclude that telomere repeat length is not significantly different between the two classes of chromosomes.

### Subtelomeric regions of core and accessory chromosomes contain the same transposable element families

**Fig. 4:**
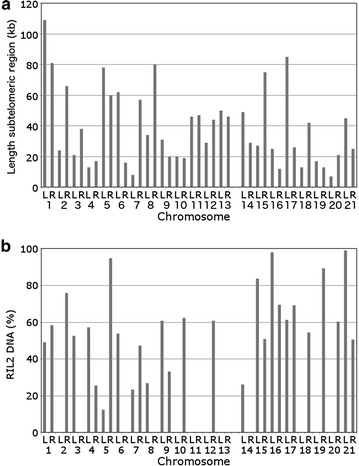
The RIL2 TE is enriched at all subtelomeres. **a** The length of subtelomeric regions is not different between core (Chr 1–13) and accessory chromosomes (Chr 14–21). **b** A family of LINE L1-like RIL2 elements is enriched in both core and accessory subtelomeric regions

Subtelomeric regions are often subject to accelerated evolution and involved in niche adaptation in microorganisms [38–41]. We analyzed *Z. tritici* subtelomeric regions to detect potential differences between core and accessory chromosomes. We found no difference in the percentage of repeat DNA (i.e., putatively active and disabled relics of TEs) at subtelomeric regions between core (83.1 %) and accessory chromosomes (84.6 %). The overall length of the subtelomeric region, defined as the longest repeat-rich region bordering the telomere repeat tracts up to the first gene-rich block, is on average only slightly longer on core chromosomes than on accessory chromosomes (43 ± 25.2 vs. 32 ± 22.3 kb, respectively). Furthermore, the same families of TEs, according to published nomenclature [12], were found in the subtelomeric regions on the core and accessory chromosomes (Additional file 11: Table S7, Additional file 12: Figure S5). The most abundant TEs or relics of TEs in the subtelomeric regions are retrotransposons (81.7 %), specifically fragments of the L1 LINE-like RIL2 elements, which are present in 34 of the 42 subtelomeric regions, for 40 % of all subtelomeric DNA (Fig. 4). Only a few DNA transposons or their relics were found in subtelomeric regions of core chromosomes and they are absent from subtelomeres of most accessory chromosomes. The DTX5 family covers 32 % of the subtelomeric region of chromosome 19L, and chromosomes 3L, 6R, 11R, 13L all have more than 10 % of their subtelomeric DNA covered by DNA transposons, but the overall total TE DNA is only 1.3 % of all subtelomeric DNA. Various non-categorized repeated DNAs (“NoCat” group) make up 8 % of the subtelomeres; this excludes simple sequence repeats. Taken together, DNA sequences at centromeres, telomere repeat tracts and subtelomeric regions show no significant differences between core and accessory chromosomes, and we therefore investigated representative histone modifications for eu- and heterochromatin at a genome-wide level.

### Subtelomeric but not centromeric chromatin is enriched with H3K9me3 and H3K27me3 on both core and accessory chromosomes

**Fig. 5:**
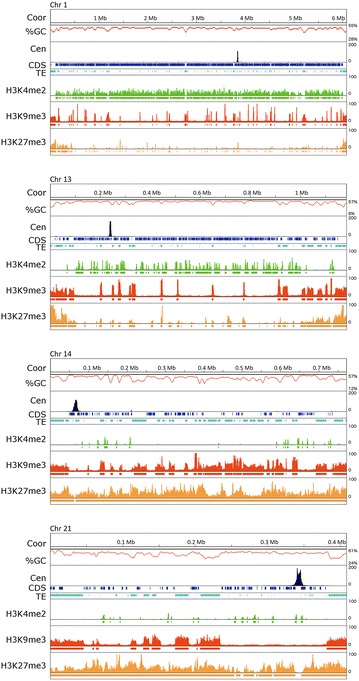
Core chromosomes are enriched with euchromatin while accessory chromosomes are enriched with heterochromatin. Core chromosomes (Chr) 1 (6.09 Mb) and 13 (1.19 Mb) are strongly enriched for H3K4me2 (*green*) and accessory Chr 14 (0.77 Mb) and 21 (0.41 Mb) are enriched for H3K9me3 (*red*) and H3K27me3 (*orange*), marks for obligate and facultative heterochromatin, respectively. There is no strong correlation between location of centromeres and enrichment for any of the histone modifications tested. H3K9me3 correlates with regions enriched for TE and H3K27me3 is found mostly in subtelomeric regions on core chromosomes. H3K27me3 is, however, found throughout most of the accessory chromosomes. For each histone modification regions with statistically significant enrichment (see “Methods” for details) are depicted by rectangles in the same color. The ruler indicates chromosome coordinates (Coor, in Mb), and GC-content (%GC, *red*), centromere position determined by GFP–CenH3 enrichment (Cen, *black*), location of coding sequences (CDS, *blue*) and active or inactive transposable elements (TE, *marine*) are shown

**Fig. 6:**
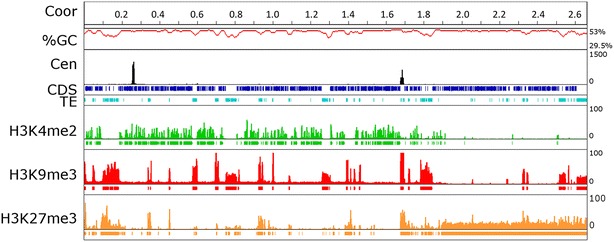
A large segment on the right arm of chromosome 7 shows similarities to an accessory chromosome. The left arm (Cen at 0.259–0.266 Mb) and most of the right arm (up to 1.8 Mb) of chromosome 7 are enriched for H3K4me2 (*green*) with H3K9me3 (*red*) and H3K27me3 (*orange*) found only in the Chr 7L subtelomeric region and TEs. A segment of ~865 kb beginning at 1.8 Mb is enriched with H3K27me3 and lacks gene expression. For each histone modification regions with statistically significant enrichment (see “Methods” for details) are depicted by *rectangles* in the *same color*. The apparent second centromeric peak and the strong H3K9me3 peak at position 1.69 Mb are explained by the presence of two rDNA repeats (see “Results” for detail). The ruler indicates chromosome coordinates (Coor, in Mb), and GC-content (%GC,* red*), centromere position determined by GFP–CenH3 enrichment (Cen, *black*), location of coding sequences (CDS, *blue*) and active or inactive transposable elements (TE, *marine*) are shown

To compare chromatin structure between core and accessory chromosomes both in centromeric regions and near telomeres, we performed ChIP-seq with antibodies against one mark for euchromatin, H3K4me2, one mark for obligate heterochromatin, H3K9me3, and one mark for facultative heterochromatin, H3K27me3 (Figs. 5, 6, Additional file 2: Table S1, Additional file 3: Figure S2). After mapping of the raw sequencing data against the reference genome of *Z. tritic*i with Bowtie2 [42], significantly enriched domains for each modification were identified with RSEG [43]. We found that locations of enriched domains in biological replicates were similar (Fig. 5, Additional file 4: Figure S3; Kendall’s Ʈ = 0.68 for H3K4me2; Ʈ = 0.88 for H3K9me3; Ʈ = 0.64 for H3K27me3; *p* < 2.2 × 10^−16^). None of the histone marks studied here were correlated with the GFP–CenH3 ChIP signal (Fig. 5). In contrast, the subtelomeric regions of all *Z. tritici* chromosomes were enriched with both H3K9me3 and H3K27me3, often in an overlapping manner (Fig. 5, Additional file 3: Figure S2) in relics of TEs or lineage-specific genes, as had been previously observed in other filamentous fungi [44–46].

### ChIP-seq reveals different distribution of histone marks on core and accessory chromosomes

As the instability of accessory chromosomes cannot be explained by differences in centromeric, telomeric or subtelomeric regions alone, we compared overall patterns of histone modifications between core and accessory chromosomes outside of centromeric and subtelomeric regions. On core chromosomes, H3K4me2 was present in ~3 kb long blocks. In contrast, H3K9me3 and H3K27me3 covered larger blocks, with an average size of more than 13 kb. Consistent with the expected positive correlation to transcriptional activity, H3K4me2 was found in genic regions near and within coding sequences (Kendall’s Ʈ = 0.44, *p* < 2.2 × 10^−16^), while H3K9me3 was found in gene-poor, repeat-rich regions (Ʈ = 0.84, *p* < 2.2 × 10^−16^; Additional file 3: Figure S2, Additional file 13: Table S8). We found a negative correlation between H3K9me3 and coding sequences (Ʈ = −0.57, *p* < 2.2 × 10^−16^). These findings were consistent between two replicates of IPO323ΔChr18. The genome-wide distribution of H3K4me2 and H3K9me3 showed that these two marks are mutually exclusive (Fig. 5). In contrast to H3K9me3, the H3K27me3 mark is located not just in repeat-rich regions but also at discrete loci in genic regions, covering promoters and coding sequences (Fig. 4, Additional file 3: Figure S2; Ʈ = −0.41, p < 2.2.10^−16^). We found a twofold enrichment of H3K9me3 on accessory chromosomes compared to core chromosomes (17 and 9 % respectively; Fig. 5, Additional file 3: Figure S2). Similarly, H3K4me2 and H3K27me3 showed very different patterns on accessory chromosomes. We found a tenfold reduction of H3K4me2 on accessory chromosomes when compared to core chromosomes (Fig. 5, Additional file 3: Figure S2; Additional file 13: Table S8), which is reflected by the much lower number of expressed genes from these chromosomes. The core chromosomes harbor 11,111 annotated genes (Additional file 14: Table S9) while all accessory chromosomes combined only have 728 genes, few of which are expressed in pure culture or even *in planta* [12, 14].

The distribution of H3K27me3 in *Z. tritici* is different from that found in other fungi [45–47]. Unlike H3K4me2 or H3K9me3, it was not clearly correlated with coding sequences or transposable elements (Additional file 13: Table S8), indicating that its distribution was broader, covering both types of sequences. H3K27me3 and H3K9me3 overlap in DNA repeats and in some gene-sized regions. In contrast, H3K27me3 and H3K4me2 are largely exclusive of each other. There is a pronounced difference in the distribution of H3K27me3 on core and accessory chromosomes: core chromosomes show blocks of H3K27me3, mostly in the subtelomeric regions, while accessory chromosomes are almost completely enriched with H3K27me3 (Fig. 5, Additional file 3: Figure S2). In summary, our genome-wide histone modification maps show that core chromosomes are largely euchromatic (enriched with H3K4me2), except in subtelomeric and other repeat-rich regions, while accessory chromosomes are largely heterochromatic (enriched with H3K9me3 and H3K27me3).

### A core chromosome segment with similarities to an accessory chromosome

The distal 0.865 Mb segment of the long right arm of chromosome 7 shows significant enrichment of H3K27me3 and near absence of the H3K4me2 mark (Fig. 6), reminiscent of the pattern observed for all accessory chromosomes. The main segment of this chromosome (1.80 Mb) has similar histone modification patterns as other core chromosomes; the centromere is localized at 259–266 kb, relatively close to the left arm telomere (TEL7L). The GC-content and gene density of the long “core” segment are at 52.5 % and 3.13 genes per 10 kb, almost identical to that of the distal “accessory” segment at 52.7 % and 3.3 genes per 10 kb, respectively. There is, however, a significant difference in gene content and organization between the two segments (Additional file 14: Table S9). The “core” segment has 563 genes with a mean gene length of 1.6 kb and half of these genes have predicted functions. The right-most, “accessory” segment has 288 genes with a mean length of 1.2 kb but only 10 % have known predicted functions. Putative secreted proteins appear to be enriched in this segment when compared to the rest of chromosome 7 (20 vs. 56 predicted secreted proteins, respectively). Thus, this region shares some characteristics with accessory chromosomes, such as almost complete absence of transcription [14] and low recombination rates [18], and we propose that this segment was translocated from an accessory chromosome or represents a fusion of a complete accessory chromosome onto the original chromosome 7. This is not without precedence, as fusions seem to have occurred in *F. oxysporum*, where the right arms of chromosomes 1 and 2 share sequence characteristics with accessory chromosomes [6]. The predicted fusion site of chromosome 7 may lie within a long AT-rich region with retrotransposon relics and near a degenerate (TTAGGG)_3_ repeat at ~1.83 Mb. Only a short distance away, at ~1.69 Mb, is the rDNA array, which in the current genome assembly only contains two repeats [14]. Between the rDNA repeats and TEL7R (nt 1,835,770–1,835,787), we found an imperfect telomere repeat (n = 3; Fig. 6). Based on genome sequencing depth of various wild-type isolates and the number of reads observed in our ChIP-seq experiments at this location (Figs. 5, 6, and data not shown), we observed 15- to 30-fold higher enrichment of reads at the two rDNA repeats compared to background coverage. Thus, we expect the rDNA cluster of *Z. tritici* to be between 30 and 60 repeats long. It remains uncertain if the locus identified on chromosome 7 is the only locus for rDNA arrays, but karyotyping in combination with Southern blots suggested only one hybridizing band to an IPO323 chromosome estimated to be 3.05 Mb long [48]. Considering that chromosome 7 is 2.67 Mb according to the current assembly [11] and that a single rDNA repeat with intergenic spacer comprises ~7 kb, ~50 rDNA repeats would yield the expected size for chromosome 7 determined by karyotyping.

## Discussion

We present the first genome-wide analysis of centromeres, telomeres, subtelomeric regions and three selected histone modifications of core and accessory chromosomes in a filamentous fungus that is also an important pathogen of wheat, and this is also the first analysis of accessory or B chromosomes by ChIP-seq. We set out to investigate potential causes for the mitotic and meiotic instability of accessory chromosomes, an enduring puzzle of general interest to chromosome biologists, by examining *Z. tritici*, a suitable model organism. Our working hypothesis stated that centromeric regions and/or different combinations of subtelomeric repeats on accessory chromosomes result in the previously observed instability [11, 17, 19]. DNA sequence, repeat content, and selected characteristics of chromatin structure revealed no significant differences between the two types of chromosomes at centromeres, subtelomeres and telomere repeat tracts. Thus, we rejected our initial hypothesis, and concluded that the instability of accessory chromosomes must be caused by other chromosome-specific traits or processes. We found one clear difference between core and accessory chromosomes in the organization of facultative heterochromatin, as assayed by ChIP-seq with antibodies to H3K27me3 nucleosomes.

The analysis of *Z. tritici* centromeres by use of GFP-tagged CenH3 revealed a novel way in which centromeres are organized. Visualization of centromere-specific fluorescence in interphase cells showed that organization of chromocenters in *Z. tritici* nuclei differs from that observed in other filamentous fungi (e.g., *F. graminearum* and *N. crassa*; [21]). In *Saccharomyces cerevisiae* [49, 50] and *Schizosaccharomyces pombe* [51, 52], as well as *Drosophila melanogaster* [53] and some plants [54], centromeres also congregate into a single chromocenter and chromosomes organize themselves into what is called the “Rabl orientation”. In *Z. tritici*, however, the GFP signal forms several discrete foci suggesting that several centromeres are located in distinct regions inside the nucleus. This is reminiscent of *Cryptococcus neoformans*, where discrete foci coalesce into one spot only upon entry into mitosis [55].

The “telomere-to-telomere” genome assembly of *Z. tritici* IPO323 allowed us to precisely identify and characterize the DNA of entire centromeric regions by ChIP-seq with GFP–CenH3. For many species assembling the centromeric DNA presents a challenge due to the high AT-content and accumulation of near-identical DNA repeats [21, 26]. Centromeric regions of *Schizosaccharomyces* species are enriched with repetitive sequences, even in the centromere cores [52]. Repeats in the centromeres of *S. pombe* and *S. octosporus* are more similar, and centromeres of *S. japonicus* are enriched with transposons in the pericentric regions [52]. In *N. crassa* centromeres are entirely composed of relics of transposable elements [27]. We show that the regional centromeres of *Z. tritici* are very short (~10.3 kb), not located in the longest AT-rich regions of the genome, and overall poor in DNA repeats. The DNA sequence for each centromere is unique and no common motif is discernible. Thus, they most resemble the short regional centromeres of *C. albicans* that also have unique DNA sequence without conserved motifs [28, 56, 57].

What makes *Z. tritici* centromeres different from other fungi is the presence of *bona fide* expressed genes. In *N. crassa*, three predicted genes were placed into centromeric DNA contigs, but all are either pseudogenes or part of a novel DNA transposon [58]. Here, we identified 39 genes that are completely within *Z. tritici* centromeric DNA or overlapping these regions. Neocentromere formation in *C. albicans* resulted in silencing of genes located within newly formed centromeres [59, 60]. Neocentromere formation in *S. pombe* occurs in regions with genes that are poorly expressed during normal growth, but induced during nitrogen starvation [61]. After neocentromere formation gene expression remains low, even after nitrogen depletion, suggesting that CenH3 nucleosomes and perhaps silencing histone marks reduce gene expression. While H3K9me3 is not required for the maintenance of centromeres and CenH3 deposition, it is required for *de novo* assembly of centromeres on plasmids [62]. In contrast to centromeres of most fungi, the much larger plant centromeres have been shown to contain genes; for example, rice centromere 8 has at least 14 predicted genes of which four are expressed [63]. A small fragment from maize chromosome 3, generated by UV mutagenesis, resulted in formation of a relatively unstable B chromosome named “Duplication 3a” (Dp3a). It carries a functional neocentromere covering 22 genes within 350 kb of a region enriched with CenH3 detected by ChIP-seq [64]. Subsequent studies further dissected the sequence requirements for this B chromosome’s neocentromere and its derivatives; composition of the DNA sequence was not a deciding factor in neocentromere formation [65]. A cross between maize and oat led to neocentromere formation, and analyses of two hybrid progeny showed 12 active genes within the newly formed centromere [66]. The functions of expressed genes in the *Z. tritici* centromeres are unknown. It is unclear if the putative alcohol dehydrogenase and aminobutyrate aminotransferase have the predicted activities.

There was no simple correlation of either euchromatic or heterochromatic histone marks with centromeric DNA. Presence of CenH3 nucleosomes did not appear to affect histone marks on canonical H3 that were tested. Like centromeres of *N. crassa* [27], but in contrast to those of *S. pombe* [67], *Drosophila* and mammals [68–70] centromeres of *Z. tritici* lack enrichment for H3K4me2. Absence of H3K4me2 has also been described for centromeric DNA of A and B chromosomes of maize [71]. Most centromeric nucleosomes of *Z. tritici* were, however, bordered by genic regions that showed H3K4me2 enrichment. H3K4me2 also surrounds the centromeres of the accessory chromosomes 19 and 21 from which the H3K4me2 mark is largely absent. In contrast to the core centromeric regions of *N. crassa* [27, 72] and *C. neoformans* [47], the centromeres of *Z. tritici* are also not enriched with the heterochromatic mark H3K9me3. In *S. pombe* and other *Schizosaccharomyces* species, the pericentric regions and to a much lesser extent the central cores are enriched with H3K9me3 [52, 62, 67]. Thus, the role of heterochromatin in centromere function first found in *S. pombe* [73, 74] and *N. crassa*, [27] is not shared in *Z. tritici*, as there are no clear pericentric heterochromatic regions. It is, however, possible that *Z. tritici* centromeres are enriched with other heterochromatic histone modifications that were not studied here such as H4K20me2, H3K27me2 or H3K9me2. In summary, by all characteristics measured here centromeres of core and accessory chromosomes are not significantly different. This suggests that interactions between centromeric DNA and some interactions between nucleosomes with centromere foundation proteins, such as CenH3, are different in *Z. tritici* when compared to other eukaryotes.

Taking all available data together, both core and accessory centromeres share some hallmarks of neocentromeres that have been found in *C. albicans*, usually after some forms of selection had been applied [59, 60, 75, 76]. So far, we have compared two closely related strains of the same *Zymoseptoria* species, both derived from the reference strain (IPO323) [11, 48]. In one strain, the *KU70* gene had been replaced (IPO323ΔKU70), the other isolate had lost chromosome 18 during culturing in the lab (IPO323Δ18). Deletion of the *KU70* gene did not result in genome instability, as one may predict when NHEJ is disabled, at least in our assays; no differences in centromere placement or other centromere features were noted. It is possible that in this relatively young and strongly host-adapted species centromeres behave differently than in species studied so far. A sexually reproducing species should, however, conserve localization of and synteny around the centromere for successful meiosis; this has been observed when comparing different species of several genera, e.g., *Aspergillus* [77], *Schizosaccharomyces* [52], *Neurospora* and *Fusarium* (M. Freitag, unpublished data). Dothideomycete chromosomes are characterized by “mesosynteny”, conserved overall chromosome structure coupled to many instances of local rearrangements [11, 78]. It is possible that these frequent reshufflings include the rather short centromeric regions to yield chromatin structure mimicking that of neocentromeres in other species. Additional *Zymoseptoria* strains and species, and additional Dothideomycetes will need to be examined to learn more about centromere positioning in these species.

Our results suggest that the reduced transmission fidelity of accessory chromosomes is not caused by differences in the structure of centromeres or telomeres. What are other quantifiable differences between core and accessory chromosomes? Commonly accessory chromosomes of fungi harbor few genes, but many active or disabled TEs [6, 11]. In *Z. tritici*, almost twice as many repetitive elements are found on accessory chromosomes [12, 79] and accessory chromosomes have much lower gene densities than core chromosomes, 1.6 *vs.* 3.2 genes per 10 kb, respectively (Additional file 14: Table S9; [12, 14]). There is ample evidence that there is little expression from accessory chromosomes, and this is certainly true for *Z. tritici* where genes on accessory chromosomes have 13-fold lower overall expression levels in both pure culture and during early host infection [12, 14]. This chromatin state correlates with the high AT and repeat content of *Z. tritici* accessory chromosomes, a correlation that holds also true in *S. pombe*, *N. crassa*, *F. fujikuroi* and *F. graminearum* [30, 46, 67, 72]. Functions for H3K9me3-enriched heterochromatic regions in fungi are still unclear, though packaging TEs and their relics into heterochromatin may prevent their spreading across the genome [72, 80]. Regulated removal of H3K9me3 may also activate genes involved in pathogenicity in *L. maculans* [81] and production of secondary metabolites in *Epichloë festucae* [82]. Outside of repeat regions, all core chromosomes have H3K27me3 enriched in subtelomeric and shorter interstitial genic regions. Accessory chromosomes, however, are almost completely covered by H3K27me3, the major difference in chromatin structure we have found that separates the two classes of chromosomes. The association of H3K27me3 with genic regions on all chromosomes suggests involvement in regulation of gene expression as observed in *Neurospora* [45], *Fusarium* [46] and *Cryptococcus neoformans* [47]. Heterochromatization is thus a quality accessory chromosomes share with the often entirely heterochromatic B chromosomes of other kingdoms [2, 7].

The H3K27me3 signal was partially overlapping with the distribution of H3K9me3, something that has so far not been found in other fungi. In *Caenorhabditis elegans*, however, both H3K9me3 and H3K27me3 can be overlapping at certain stages of development and in certain regions of the genome [83], though this was not obvious in an earlier study [84]. Overlapping H3K9me3 and H3K27me3 was generated in centromeric domains by mutating *C. neoformans Ccc1* [47], a protein with H3K27me3-binding activity that is required to maintain H3K27me3 in specific subtelomeric regions. There is also precedence for shifts in H3K27me3 from studies in plants [85] and mammals [86], where H3K27me3 moved into regions covered by H3K9me3 when cytosine DNA methylation had been removed by mutation of DNA methyltransferase genes. The reference strain of *Z. tritici* is deficient in cytosine DNA methylation because the conserved homolog of *N. crassadim*-*2*, *MgDnmt*, underwent gene duplication to more than 20 copies followed by inactivation by Repeat Induced Point mutation (RIP) [87]. Thus, we hypothesize that the overlapping H3K9me3 and H3K27me3 we observed is a consequence of the lack of DNA methylation, similar to the results from plants and mammals.

Many studies have shown that nuclear position of chromosome segments can affect gene expression [88–90]. Based on the analysis of centromere chromocenters (by GFP–CENH3 microscopy) and chromatin structure (distribution of H3K4, H3K9, and H3K27 methylation by ChIP-seq), our study allows us to propose the existence of at least two different regions of chromosome organization in *Z. tritici* nuclei. Accessory chromosomes are almost entirely heterochromatic, similar to B chromosomes in other eukaryotes, while core chromosomes are mostly euchromatic. Studies on stretched chromatin fibers of maize stained with antibodies against various histone modifications suggested depletion of H3K27me2 from both B chromosomes [71]. While H3K27me3 was not examined in that study, H3K9me2, a well-studied indicator of heterochromatin, was enriched on B chromosomes. Core chromosomes of both *Zymoseptoria* and maize are mainly euchromatic, in *Z. tritici* with some relatively short interspersed heterochromatic regions and larger blocks of heterochromatin at the chromosome ends. In *S. cerevisiae* Rabl orientation is maintained in interphase as revealed by HiC-based modeling of the nucleus [91]. Strong centromeric and pericentric interactions have also been demonstrated in *S. pombe* [92] and *Arabidopsis* [93] HiC chromatin maps. In several studies, domains enriched with H3K27me3 have been shown to form blocks of chromatin that may condense into “polycomb bodies” and appear to be located, if not anchored, near the nuclear membrane [83, 94, 95]. Based on our studies, we formulated a testable working model for chromatin architecture in *Z. tritici*, in which interactions between centromeres of the 13 core chromosomes generate one main chromocenter, while interactions of H3K27me3-rich chromatin of the eight accessory chromosomes form “silencing bodies” near the nuclear membrane. This implies that for accessory chromosomes interactions between H3K27-methylated chromosome arms are stronger than interactions that bring centromeric regions of different accessory chromosomes together. These H3K27me3 domains may thus “trap” and separate centromeres of accessory chromosomes in the nucleus, resulting in the additional, weakly fluorescing chromocenters. Work on maize also suggested that less CenH3 is incorporated at centromeres on plant B chromosomes, resulting in weaker CenH3 immunofluorescence [71]. One prediction of our hypothesis was that comparisons between GFP–CenH3 strains with a full chromosome complement and those that lack chromosome 18 would reveal differences in the average number of foci. Preliminary experiments suggest, however, that the situation is not as simple as proposed above because the median number of foci observed is not different. Instead, we saw minor changes in the number of bright foci. Future experiments using cytological and chromosome conformation capture approaches will be applied to further test our hypothesis.

## Conclusions

We hypothesized that core and accessory chromosomes have distinct centromeres and that the centromeric organization leads to meiotically unstable accessory chromosomes. Besides the relative location of centromeres (acrocentric on most core, metacentric on most accessory chromosomes), however, there are no obvious measurable differences. Centromeres of *Z. tritici* are not perfectly associated with either canonical heterochromatin or euchromatin and they contain genes that are overall poorly expressed. Overall they exhibit features that have been considered common for neocentromeres in other organisms. Moreover, there is no significant difference between telomeric repeat sequences, repeat length and composition of the adjacent subtelomeric TEs on core and accessory chromosomes. The right arm of core chromosome 7 is poorly transcribed and enriched with heterochromatin. Based on these criteria, otherwise only found for accessory chromosomes, we propose that an accessory chromosome was fused to a core chromosome, resulting in the extant chromosome 7. We show that accessory chromosomes of *Z. tritici* can be distinguished from core chromosomes based on enrichment with H3K27me3. Whether this enrichment with a mark for facultative heterochromatin is causally involved in the reduced transmission fidelity of accessory chromosomes will be the focus of future work.

## Methods

### Strains and growth conditions

All experiments were performed with derivatives of the *Z. tritici* strain used for the reference genome, IPO323 [11]. In IPO323ΔChr18 (Zt9), chromosome 18 has been lost [14, 48], and in IPO323ΔKU70 (Zt84), the *KU70* gene has been disrupted to increase the efficiency of homologous recombination [29]. That our original IPO323 isolate had lost chromosome 18 was discovered in the course of studies described here. Streaks from glycerol stocks (kept at −80 °C) were used as initial inoculum on YMS (4 g yeast extract, 4 g malt extract, 4 g sucrose, 20 g agar per 1 L H_2_O) agar plates. Cultures were grown for four to six days at 18 °C. Cells were transferred to liquid YMS medium and grown for 3 days at 18 °C while shaking at 200 rpm.

### DNA extraction

DNA was extracted from cells by glass-bead homogenization using a previously described phenol–chloroform method [96]. Genomic DNA was used for PCR and Southern analyses according to previously published protocols [97].

### Construction of GFP-tagged strains

The homolog of CenH3, previously described as centromere-specific protein in other organisms [21, 23], was identified in the *Z. tritici* genome (http://genome.jgi-psf.org/Mycgr3/Mycgr3.home.html; [11]) by BLAST analyses [31] with *N. crassa* CenH3 [27]. To introduce the GFP epitope tag, we designed constructs for homologous gene replacement based on the binary vector D0893pNOVpGpda SDHB_H267YtTrpC, which carries *hph*, the gene encoding resistance to Hygromycin B [29]. We amplified the predicted open reading frame of the CenH3 gene, as well as 1 kb of 5′ and 3′ flanking sequences of each gene from genomic DNA of strain Zt9, and the GFP tag from pZero-GFP-loxP-hph-loxP [98] but with a 6X glycine linker. All primers are listed in Additional file 15: Table S10. Purified PCR amplified fragments were fused by overlap-PCR [99] to create a construct consisting of all fragments. The new construct was inserted into the binary vector at the unique Bsp120I and AscI sites. Plasmid sequences were verified by restriction analyses and Sanger sequencing. Constructs were introduced into Zt9 and Zt84 by *Agrobacterium tumefaciens*-mediated transformation [97], but with minor modifications. Correct insertions of the replacement cassettes were determined by an initial PCR screen followed by Southern analysis on positive clones [97]. Isogenic strains resulting from transformation with GFP–CenH3 in Zt9 are called Zt121 (isolates Zt121-57 and Zt121-85) and those in Zt84 are called Zt118 (Zt 118-5 and Zt118-7).

### Western analysis

Western analyses were done to verify translation of the GFP-tagged CenH3 protein. Proteins were extracted from GFP–CenH3 strains Zt118 and Zt121 by the peqGOLD TriFast protocol (Peqlab, Erlangen, Germany). Prior to electrophoresis, protein levels were normalized by Bradford assay [100]. Western blotting was done by standard methods [96]. Detection of the GFP signal was using the ECL prime kit (GE Healthcare Europe GmbH, Freiburg, Germany) according to instructions of the manufacturer. Anti-GFP antibody (Roche; #11 814 460 001) and secondary anti-mouse IgG-HRP antibody (Cell Signaling; #7076 G) were used.

### Fluorescence microscopy

To assess localization of GFP-tagged centromere proteins, we used fluorescence microscopy on a Zeiss Axioplan 2 Microscope System equipped with a 100X oil immersion objective (1.4 N/A); standard excitation filters and emission filters for detection of GFP fluorescence were used. Fluorescence micrographs were taken with a Cool SNAP HQ camera (Photometrics) and manipulated in Gimp (Version 2.8.10). To assess the number of GFP foci, numerous still images were taken of strains Zt121 (IPO323ΔChr18) and Zt118 (IPO323ΔKU70), both producing GFP–CenH3. Bright and weak GFP foci in nuclei were counted to determine if Zt121 had on average fewer foci than Zt118.

### Chromatin immunoprecipitation followed by high-throughput sequencing (ChIP-seq)

ChIP experiments were performed on the yeast-like stage after growth in liquid YMS medium for 4 days until an OD_600_ of 0.6–0.8 had been reached. ChIP experiments were performed as previously described [27, 101] with minor modifications [102]. Histones with the modification of interest were immunoprecipitated by adding 2 μL of antibodies to ~300–500 μL of purified chromatin. ChIP was performed with the following antibodies: anti-GFP (#ab290; Abcam, Cambridge, MA, USA), anti-H3K4me2 (#07-030; Millipore, Billerica, MA, USA), anti-H3K9me3 (#39161; Active Motif, Carlsbad, CA, USA) and anti-H3K27me3 (#39155; Active Motif, Carlsbad, CA, USA). Libraries for ChIP-seq were prepared according to Illumina TruSeq protocols with some modifications and controls [102, 103]. Purification of DNA was performed with AMPure XP beads (Agencourt, Beckman-Coulter). To size select samples were gel-purified and PCR amplified by 13 PCR cycles with Phusion polymerase (Finnzymes) and Illumina PCR primers. Libraries were sequenced on an Illumina HiSeq 2000 genome analyzer at the OSU Center for Genome Research and Biocomputing. Sequencing data were submitted to SRA under accession number SRP059394. ChIP tracks are viewable on a dedicated gbrowse server at http://ascobase.cgrb.oregonstate.edu/cgi-bin/gb2/gbrowse/ncrassa_public/.

### Short read mapping and peak calling analyses

Illumina reads were filtered and trimmed as previously described [14], but they were not trimmed from the 5′ end, resulting in 47-nt reads. Processed reads were mapped to the *Z. tritici* IPO323 reference genome [11] with Bowtie2 at standard settings [42]. Mapping outputs were converted from “.sam” to “.bed” files using Samtools and Bedtools [104, 105]. For visualization of mapped reads, alignment files from Bowtie2 were either processed with the genomecov tool from the bedtools package [105] and the Integrative Genome Viewer (IGV; http://www.broadinstitute.org/software/igv); [106] or loaded on a dedicated public gbrowse server (http://ascobase.cgrb.oregonstate.edu/cgi-bin/gb2/gbrowse/ncrassa_public/). For each ChIP sample (Additional file 2: Table S1), we determined significantly enriched domains with RSEG [43]. Two biological replicates were also evaluated separately to assess variability between ChIP-seq experiments. The coverage or relative enrichment of coding sequences, repetitive elements and histone modifications on each chromosome were calculated in non-overlapping sliding windows of 10 kb. For correlation analyses in R (http://www.R-project.org), we used Kendall’s Tau correlation coefficient [107].

### Identification of AT-rich regions

AT-rich regions were identified in the genome assembly of *Z. tritici* IPO323 using a homemade python script that computed the local GC-content in 1 kb sliding windows with a shift of one bp. AT-rich regions were defined as a concatenation of consecutive windows having a GC-content value lower than 50 %.

## Additional files

10.1186/s13072-015-0033-5 Confirmation of GFP–CenH3 strains by Southern and western analyses. A. Map of the CenH3 locus before (top) and after (bottom) transformation. B. Southern analysis shows the correct integration of the GFP–CenH3 construct. Genomic DNA was digested with NcoI. DNA from IPO323∆Chr18 (Zt9) and IPO323∆KU70 (Zt84) shows a single band of 7,327 bp (see A). The purified transformants (Zt121-57, Zt121-85, Zt118-5 and Zt118-7) with the correctly integrated GFP–CenH3 gene show two expected bands at 5,455 and 4,014 bp. The downstream flank (DF; primers pES709 and pES710) and upstream flank (UF; pES715 and pES716) were used as mixed probes. C. Western analysis shows the correct translation of the GFP–CenH3 protein in the GFP–CenH3 IPO323ΔChr18 (Zt121-57 and Zt121-85) and GFP–CenH3 IPO323ΔKU70 (Zt118-5 and Zt118-7) isolates. A positive control (IPO323_ZT77228-D::chr1ncr_nat.Pr.77228GFP, Zt144) was included. As expected IPO323ΔChr18 (Zt9) and IPO323ΔKU70 (Zt84) lacked signals for the GFP–CenH3.
10.1186/s13072-015-0033-5 Statistics of ChIP-seq experiments. Number of total reads after filtering and mapping is shown. Percentage of reads mapping uniquely, in more than one position or that remained unmapped is based on the total amount of reads. Data were submitted to SRA under accession number SRP059394.
10.1186/s13072-015-0033-5 Chromosomal overview with centromeric position and histone modifications. Full length chromosomes from Zt121-1 (GFP–CenH3 in IPO323ΔChr18) are shown (drawn to scale). The ruler indicates the length of the chromosomes. For each chromosome the GC-content (%GC, red), centromeric position (Cen, black), gene density (CDS, blue), and TE content are shown (TE, marine). Enrichment with H3K4me2 (green), H3K9me3 (red) and H3K27me3 (orange) is shown.
10.1186/s13072-015-0033-5 Centromere view with centromeric position and histone modifications for all chromosomes. Centromeric regions from Zt121-1 (GFP–CenH3 in IPO323ΔChr18) are shown. The ruler indicates the length of the chromosomes. For each chromosome the GC-content (%GC, red), centromeric position (Cen, black), gene density (CDS, blue), and TE content are shown (TE, marine). Enrichment with H3K4me2 (green), H3K9me3 (red) and H3K27me3 (orange) is shown.
10.1186/s13072-015-0033-5 Primers and length of the amplification products to verify the length of the centromeres. For each centromere, three PCR reactions were performed. PCR reaction 1 amplifies the whole centromeric region, and PCR reactions 2 and 3 amplify two parts of the centromeric region. Primer IDs, primers sequences and the expected product are shown for all PCR amplifications.
10.1186/s13072-015-0033-5 Core chromosomes of *Z. tritici* are mostly acrocentric or near-acrocentric while accessory chromosomes are mostly metacentric. A. Diagram outlining the definitions of metacentric and acrocentric used here. Metacentric is the middle third of the chromosomes (falling between 33 and 50 % of chromosome length counted from either telomere). Near-metacentric is chromosomes from 20 to < 33 %, near-acrocentric is chromosomes from 10 to < 20 %, and acrocentric is chromosomes from > 0 to < 10 % of chromosome length counted from either telomere. B. Core chromosomes (Chr 1 to 13) are mostly acro- or near-acrocentric, while accessory Chr 14 to 21 are mostly metacentric. Green shading indicates relative position of centromeric region on chromosomes as shown in A. The x-axis shows the chromosome numbers, the y-axis shows the relative position of the centromere on each chromosome based on the diagram in A.
10.1186/s13072-015-0033-5 Centromeres are not located in the longest AT-rich region of the chromosome. A. The number of AT-rich regions in all chromosomes, including the length of the AT-rich region, the coordinates of the AT-rich regions and the percentage of repetitive DNA (mostly TEs) are listed. B. Number of AT-rich regions in all centromeres, including the length of the AT-rich region, the coordinates of the AT-rich regions and the percentage of repetitive DNA (mostly TEs). In total there are 847 AT-rich regions, from 2 kb to 86.3 kb in size and covering 7.383 Mb of the genome. The median size is 7.1 kb and the mean AT-content is 55 %. In chromosomes 4 and 10 two AT-rich regions partially overlap the centromeres, on chromosome 4 this is 1.905 kb of 86.31 kb and on chromosomes 10 this is 1.77 kb of 11.8 kb; these regions are indicated by an asterisk (*).
10.1186/s13072-015-0033-5 Repetitive regions and annotated transposable elements (TEs) in the centromeres. Position of repeats (Start and Stop) is relative to centromere positions (Table 1; Cen Start and Stop). Categorized TE-families are labeled according to the nomenclature proposed by Wicker and colleagues [34]. “NoCat” means that repetitive DNA does not match TE sequences.
10.1186/s13072-015-0033-5 Putative genes in centromeric regions and their expression. In total, 39 putative genes were located within or overlapping centromeric regions. Genes with a mean RPKM value > 2 are considered expressed (indicated by √). Expression data were from a previous study [14] and both the original JGI [11] and our new gene IDs [12] are shown. Expression levels for control genes (GAP-DH, histone H3, beta-tubulin and CenH3) are shown for comparison. The mean expression level of centromeric genes on core chromosomes was 385.4 ± 1881.1 and on accessory chromosomes 1.72 ± 2.8.
10.1186/s13072-015-0033-5 Length of telomeric repeat sequences on core and accessory chromosomes. The length of the telomere repeat tracts (TTAGGG)_n_ was determined from the available genome sequence. As sequencing into chromosome ends leaves some uncertainty about the true length of telomeres in *Z. tritici*, these data are included to show that there are no significant differences between the currently available sequence from core and accessory chromosomes. Near TTAGGG repeat sequences abutting the telomere tracts are also shown. Overall, core chromosomes have slightly longer telomeres than accessory chromosomes (133 bp *vs.* 118 bp) with 2.5 additional repeats (22.3 *vs.* 19.8 repeats/telomere).
10.1186/s13072-015-0033-5 Repetitive DNA at the subtelomeric regions. Percentage of TE-families and repetitive elements is shown for each subtelomeric region. The length of the subtelomeric region, TE-families/repeats with a coverage > 10 % and the total amount of TE/repeats (%) are shown. Categorized TE-families are labeled according to nomenclature proposed recently [34]. “NoCat” means that repetitive DNA does not match TE sequences.
10.1186/s13072-015-0033-5 Subtelomeric regions of Z. tritici core (Chr 1 to 13) and accessory (Chr 14 to 21) chromosomes contain the same families of repetitive elements and TEs. Repeat families are labeled as described previously [12]. RIL 2 repeats make up the majority of all subtelomeric elements on both chromosome types (see also Table S5).
10.1186/s13072-015-0033-5 Correlation analyses of ChIP-seq data obtained for different histone modifications. Correlation analyses were performed on coverage data along core or accessory chromosomes alone and on all chromosomes combined. The coverage of transposable elements (TE) or coding sequences (CDS) for each histone modification (H3K4me2, H3K9me3, H3K27me3) was considered. Two biological replicates (Replicate 1 and 2) were generated and analyzed separately. The Kendall’s Tau correlation test was applied [107], as provided in the R statistical package.
10.1186/s13072-015-0033-5 Organization of chromosome 7. A. Genes on core and accessory chromosomes according to our updated annotation [12]. Overall gene density is clearly lower for accessory compared to core chromosomes. B. Same analyses for the whole chromosome 7, the core segment (Tel7L to 1.8 Mb) and the right, accessory segment (1.8 Mb to Tel7R). Even though the right-most segment shows hallmarks of an accessory chromosome gene density is higher than on the left segment.
10.1186/s13072-015-0033-5 Primers used for construction of plasmids in this study. Primer ID and primer sequence used for construction of the GFP–CenH3 plasmid are shown.

## Abbreviations

AT-rich: adenine and thymine rich
CenH3: centromere-specific histone H3 (CENP-A of mammals)
ChIP-seq: chromatin immunoprecipitation followed by massively parallel DNA sequencing
Chr: chromosome
GFP: green fluorescent protein
H3K4me2: dimethylated histone H3 lysine 4
H3K9me2: dimethylated histone H3 lysine 9
H3K9me3: trimethylated histone H3 lysine 9
H3K27me2: dimethylated histone H3 lysine 27
H3K27me3: trimethylated histone H3 lysine 27
H4K20me2: dimethylated histone H4 lysine 20
HiC: chromosome conformation capture followed by massively parallel sequencing
kb: kilobase
Mb: megabase
NHEJ: non-homologous end joining
NoCat: unclassified DNA repeat
RPKM: reads per kilobase of transcript per million mapped reads
TE: transposable element
YMS: yeast malt sucrose
