# Supplementary figures and images for: Histone modifications rather than the novel regional centromeres of Zymoseptoria tritici distinguish core and accessory chromosomes
Source: Epigenetics Chromatin. 2015 Oct 1;8:41. doi: 10.1186/s13072-015-0033-5 (PMC4589918; doi:10.1186/s13072-015-0033-5)

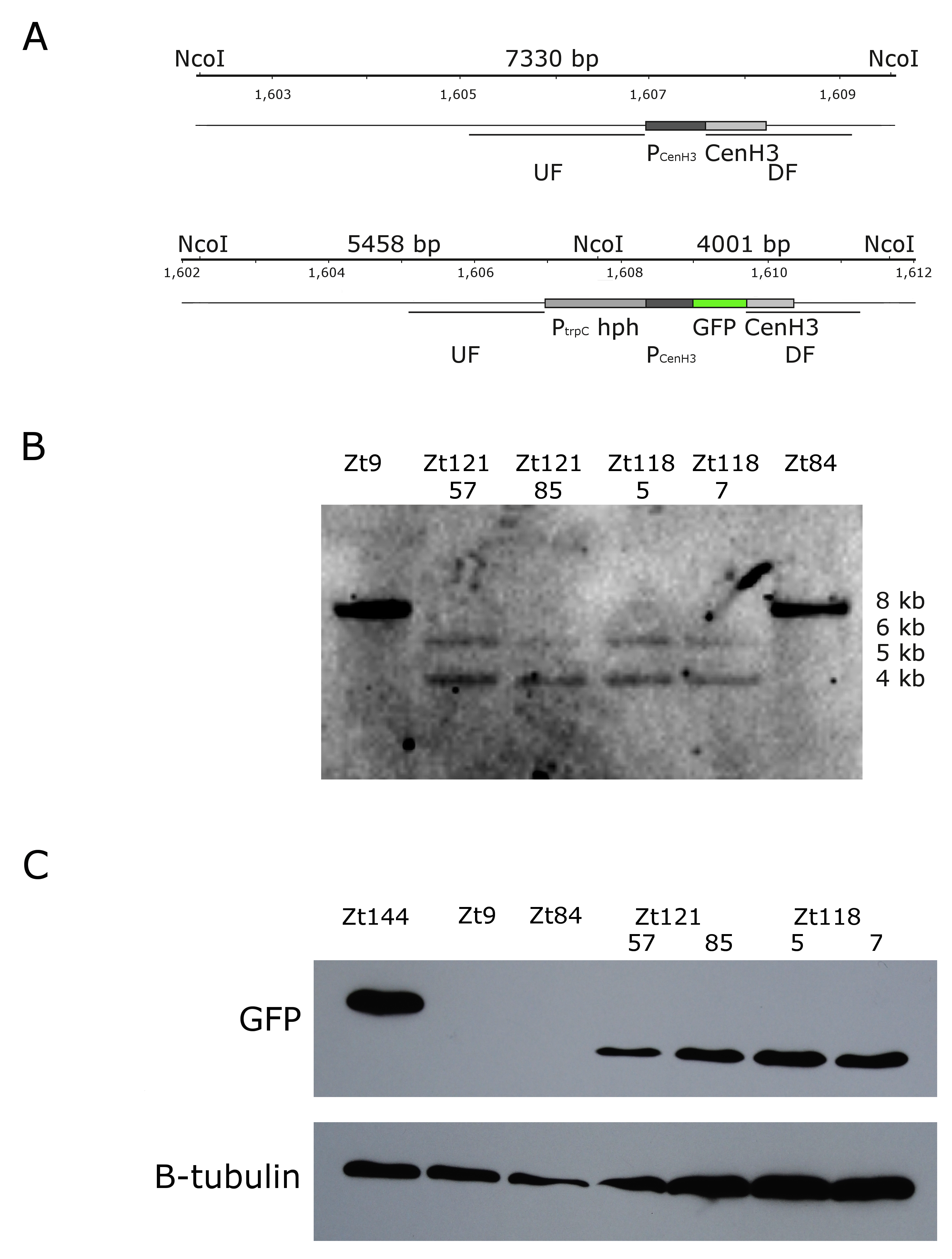

Supplement: Supplementary file 1 — 10.1186/s13072-015-0033-5 Confirmation of GFP–CenH3 strains by Southern and western analyses. A. Map of the CenH3 locus before (top) and after (bottom) transformation. B. Southern analysis shows the correct integration of the GFP–CenH3 construct. Genomic DNA was digested with NcoI. DNA from IPO323∆Chr18 (Zt9) and IPO323∆KU70 (Zt84) shows a single band of 7,327 bp (see A). The purified transformants (Zt121-57, Zt121-85, Zt118-5 and Zt118-7) with the correctly integrated GFP–CenH3 gene show two expected bands at 5,455 and 4,014 bp. The downstream flank (DF; primers pES709 and pES710) and upstream flank (UF; pES715 and pES716) were used as mixed probes. C. Western analysis shows the correct translation of the GFP–CenH3 protein in the GFP–CenH3 IPO323ΔChr18 (Zt121-57 and Zt121-85) and GFP–CenH3 IPO323ΔKU70 (Zt118-5 and Zt118-7) isolates. A positive control (IPO323_ZT77228-D::chr1ncr_nat.Pr.77228GFP, Zt144) was included. As expected IPO323ΔChr18 (Zt9) and IPO323ΔKU70 (Zt84) lacked signals for the GFP–CenH3. [file 13072_2015_33_MOESM1_ESM.tif]

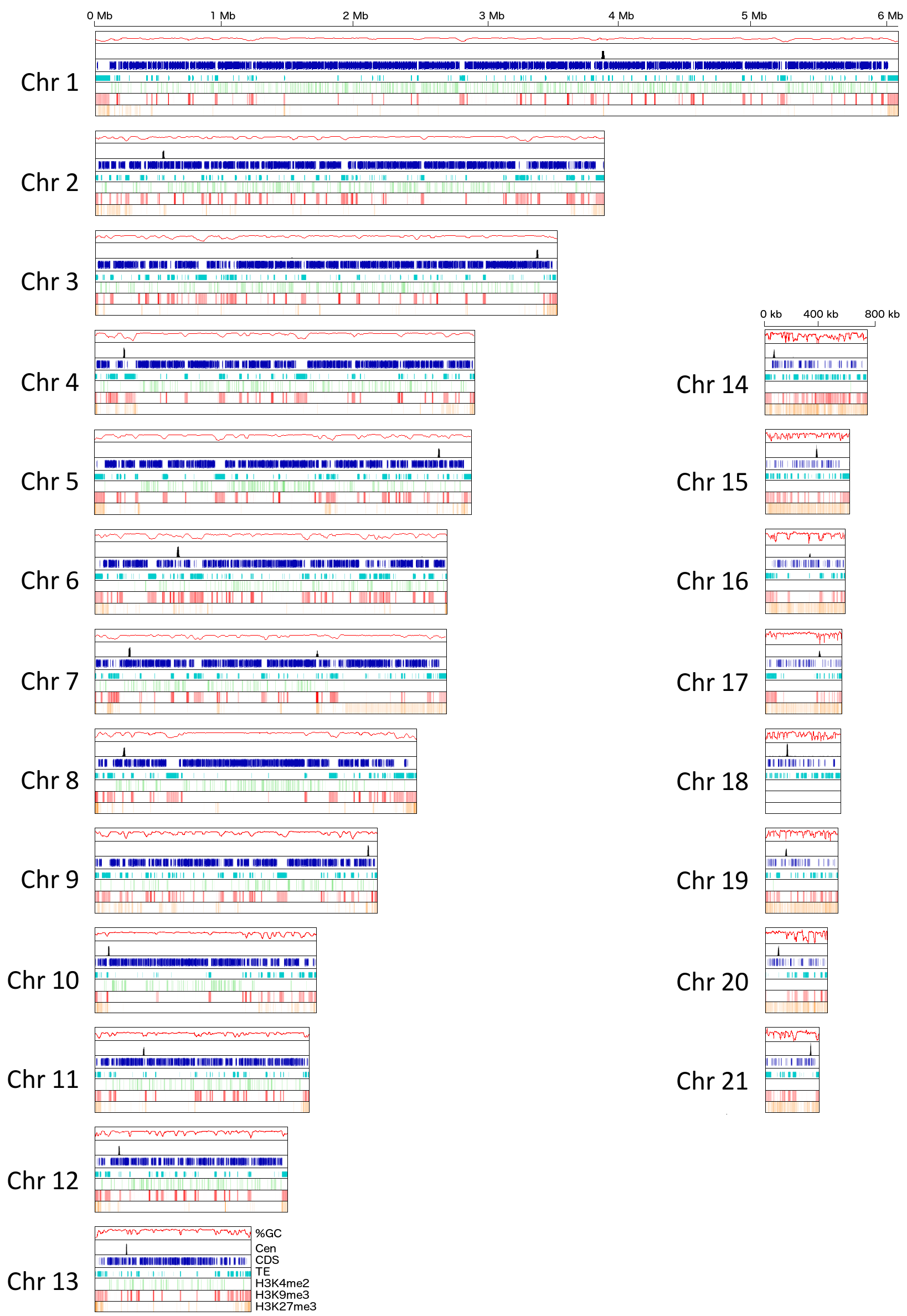

Supplement: Supplementary file 3 — 10.1186/s13072-015-0033-5 Chromosomal overview with centromeric position and histone modifications. Full length chromosomes from Zt121-1 (GFP–CenH3 in IPO323ΔChr18) are shown (drawn to scale). The ruler indicates the length of the chromosomes. For each chromosome the GC-content (%GC, red), centromeric position (Cen, black), gene density (CDS, blue), and TE content are shown (TE, marine). Enrichment with H3K4me2 (green), H3K9me3 (red) and H3K27me3 (orange) is shown. [file 13072_2015_33_MOESM3_ESM.tif]

Chr 1

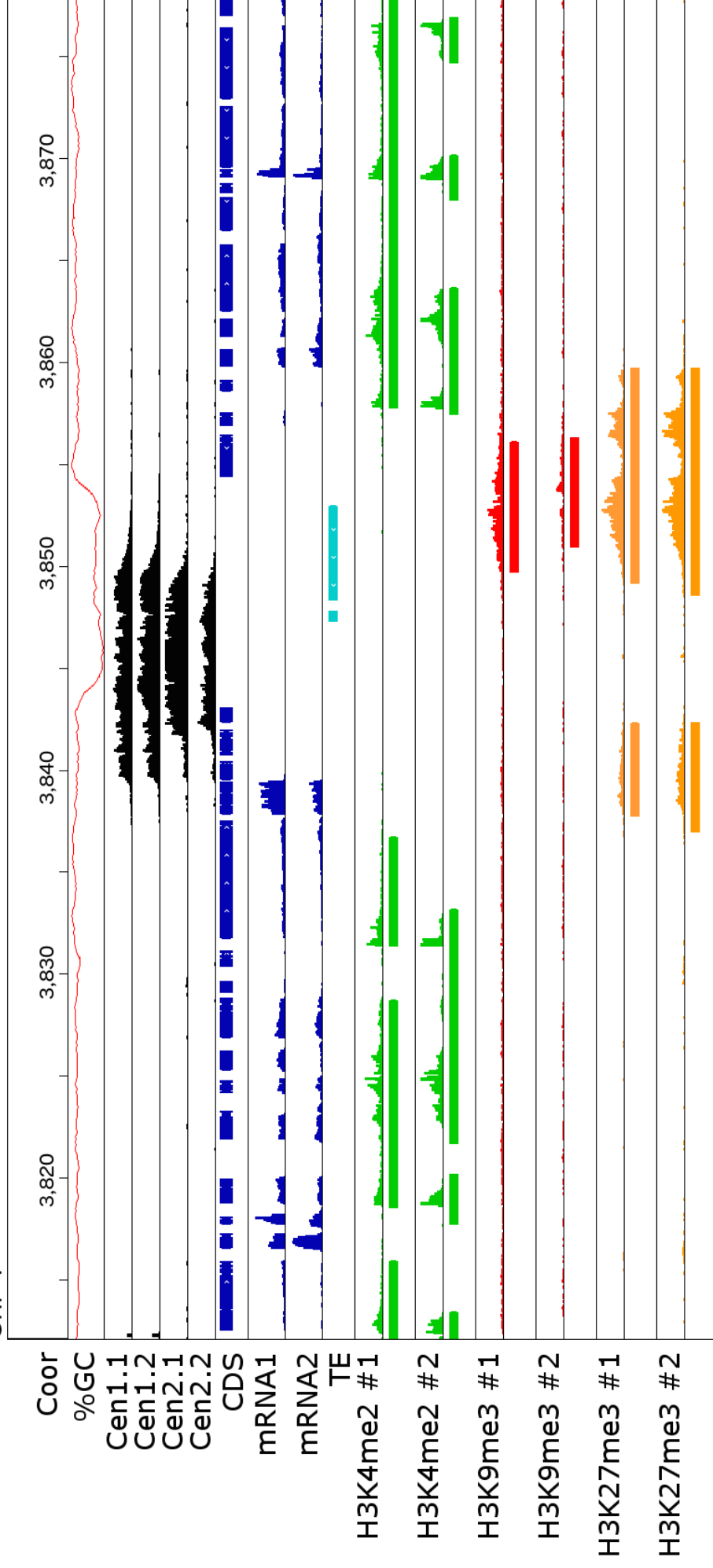

Chr 2

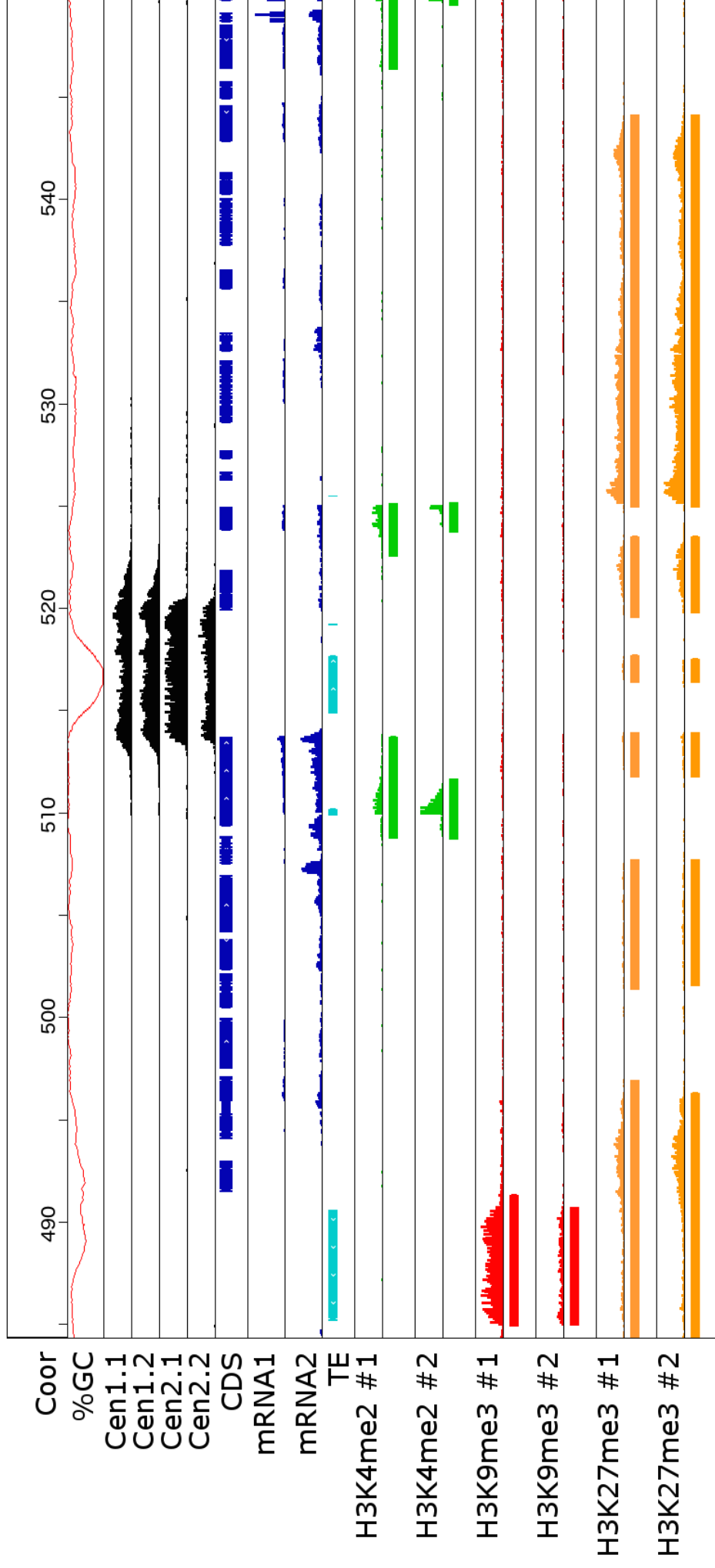

Chr 3

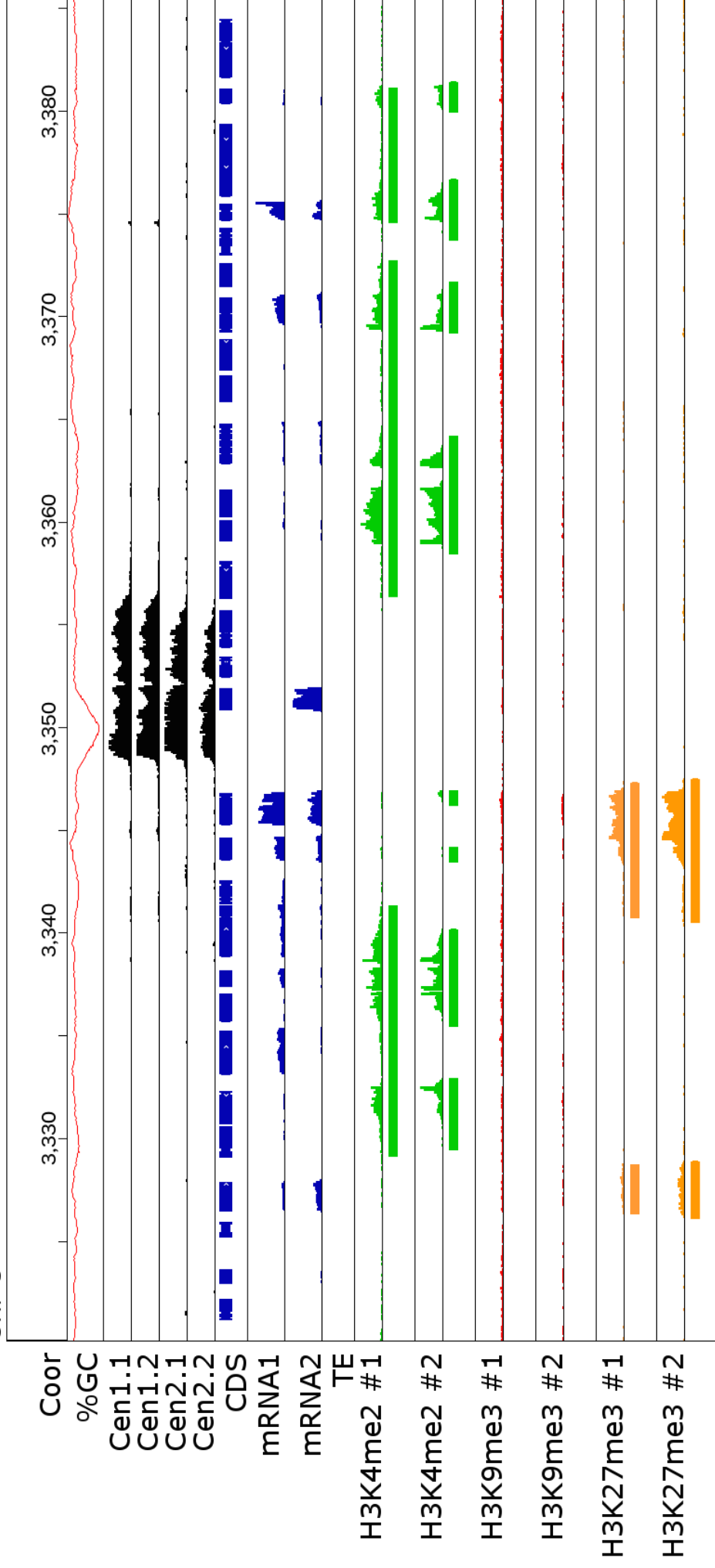

Chr 4

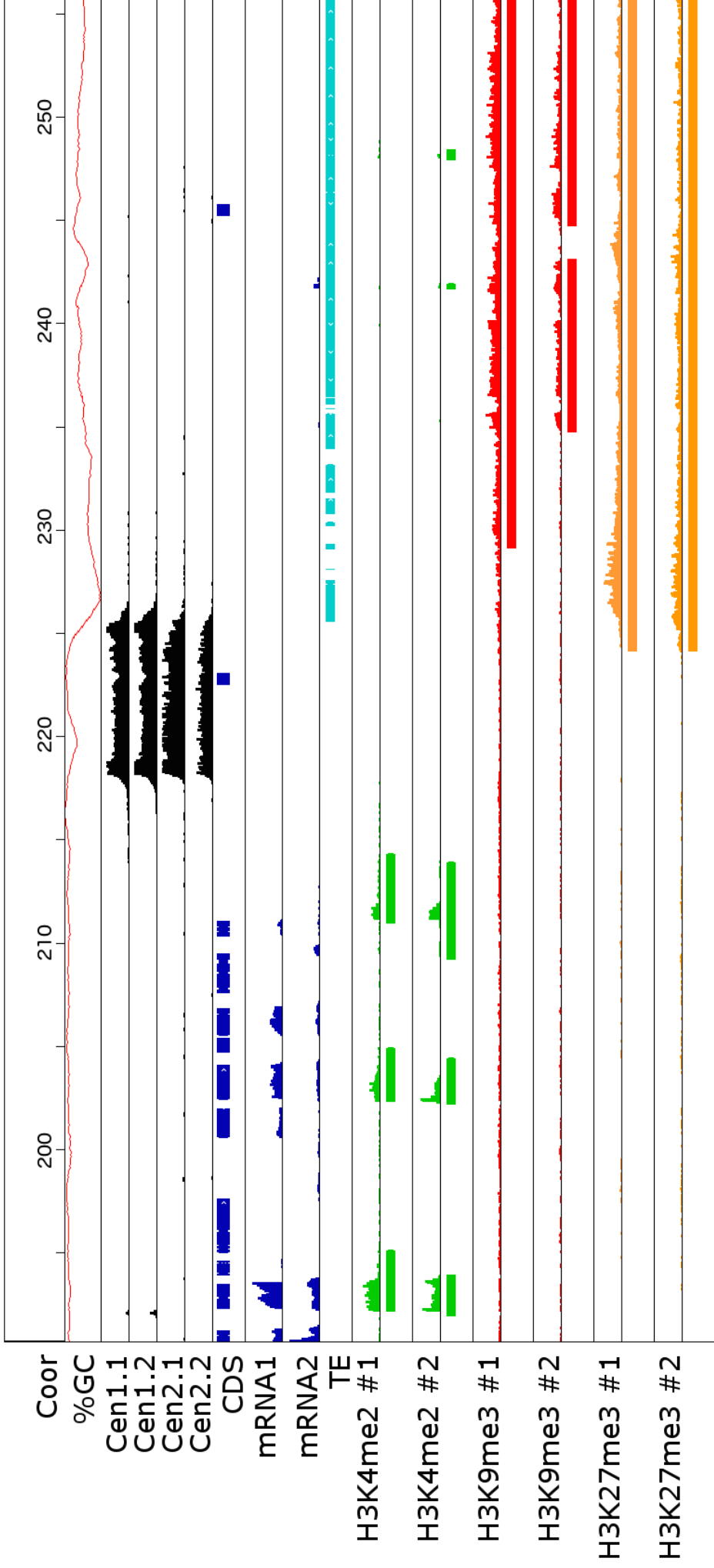

Chr 5

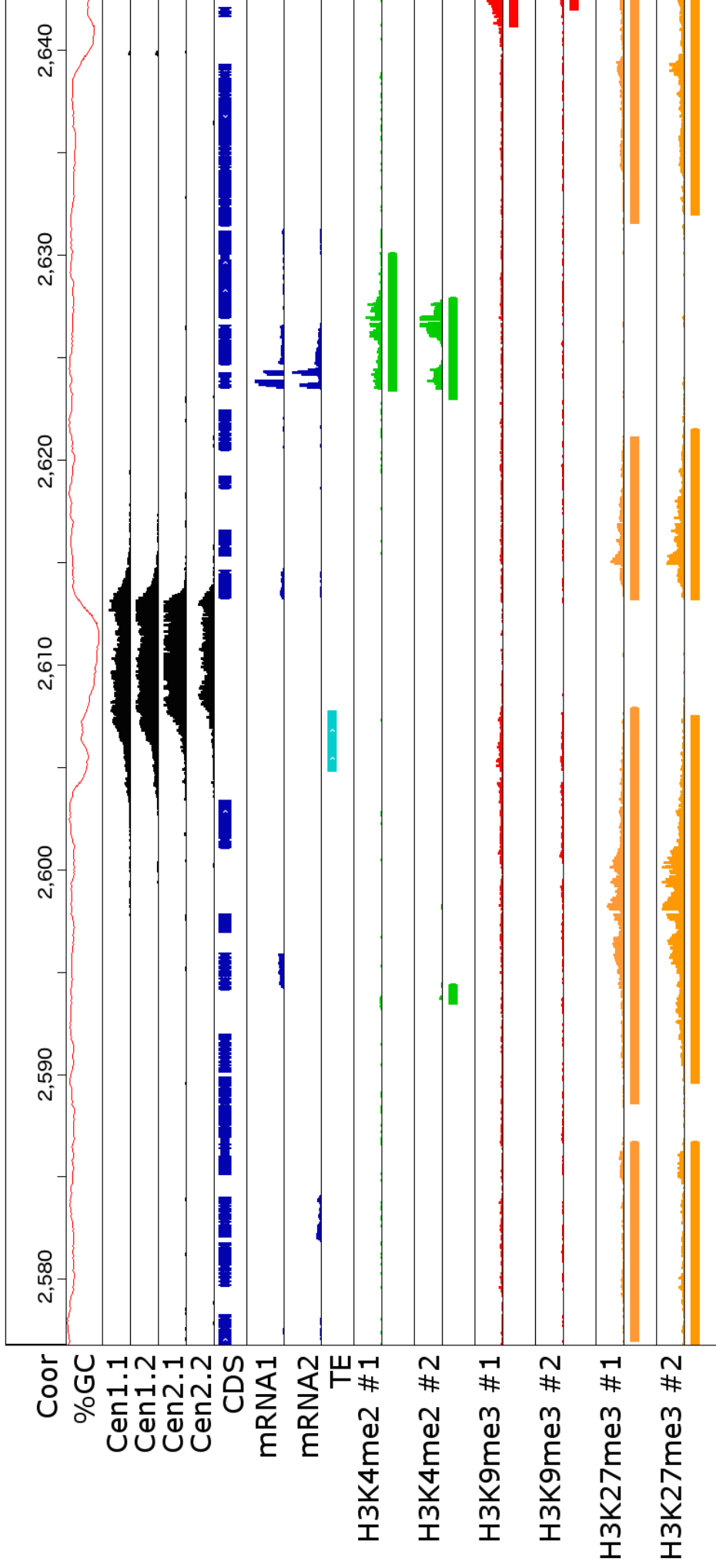

Chr 6

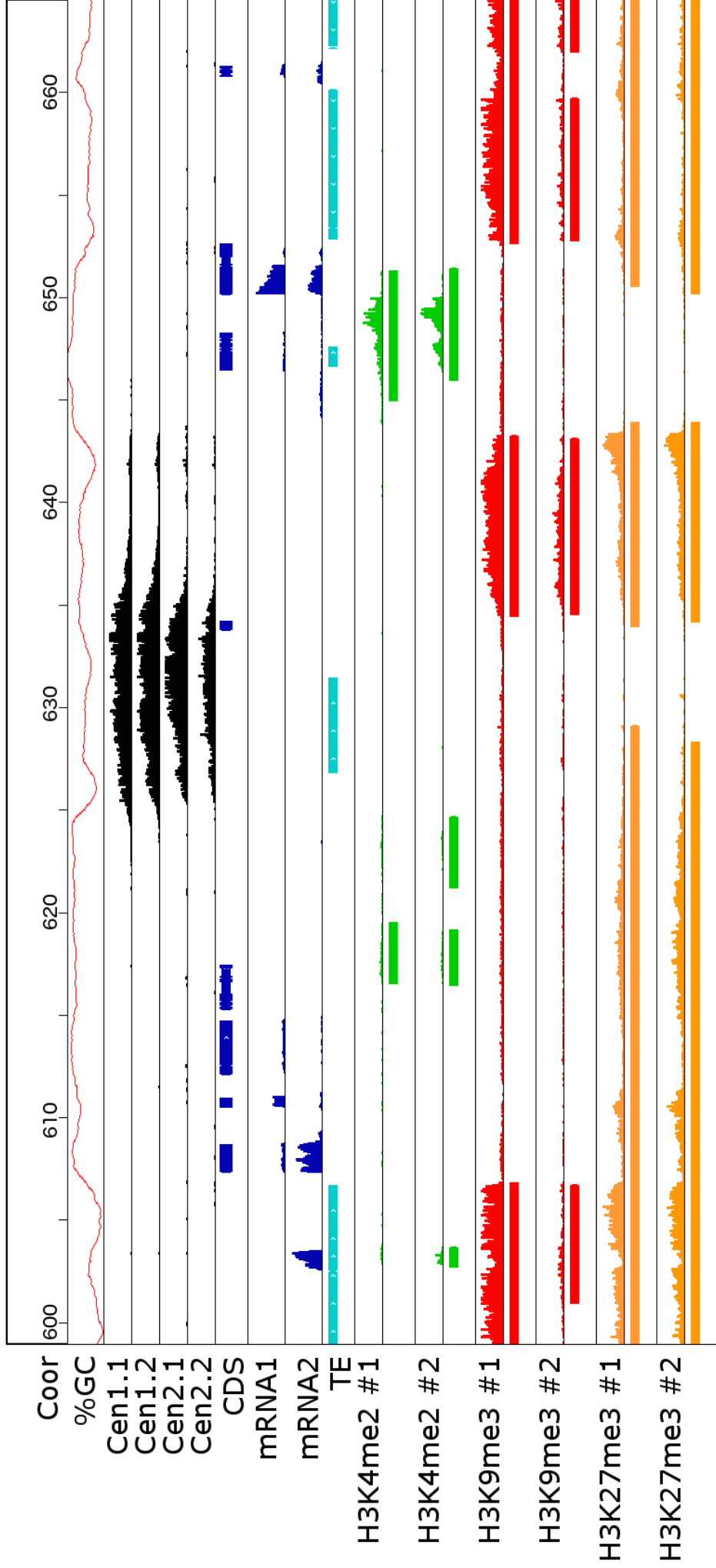

Chr 7

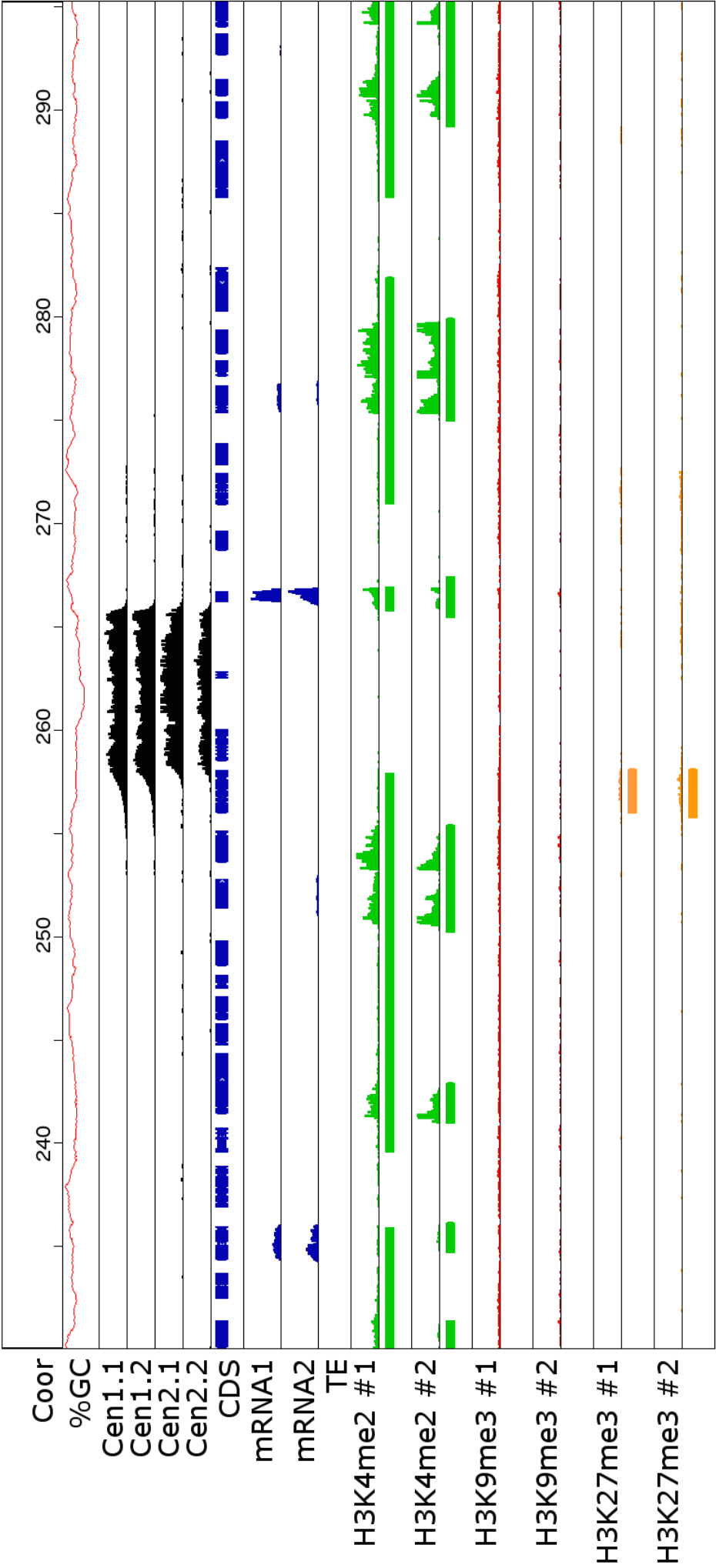

Chr 8

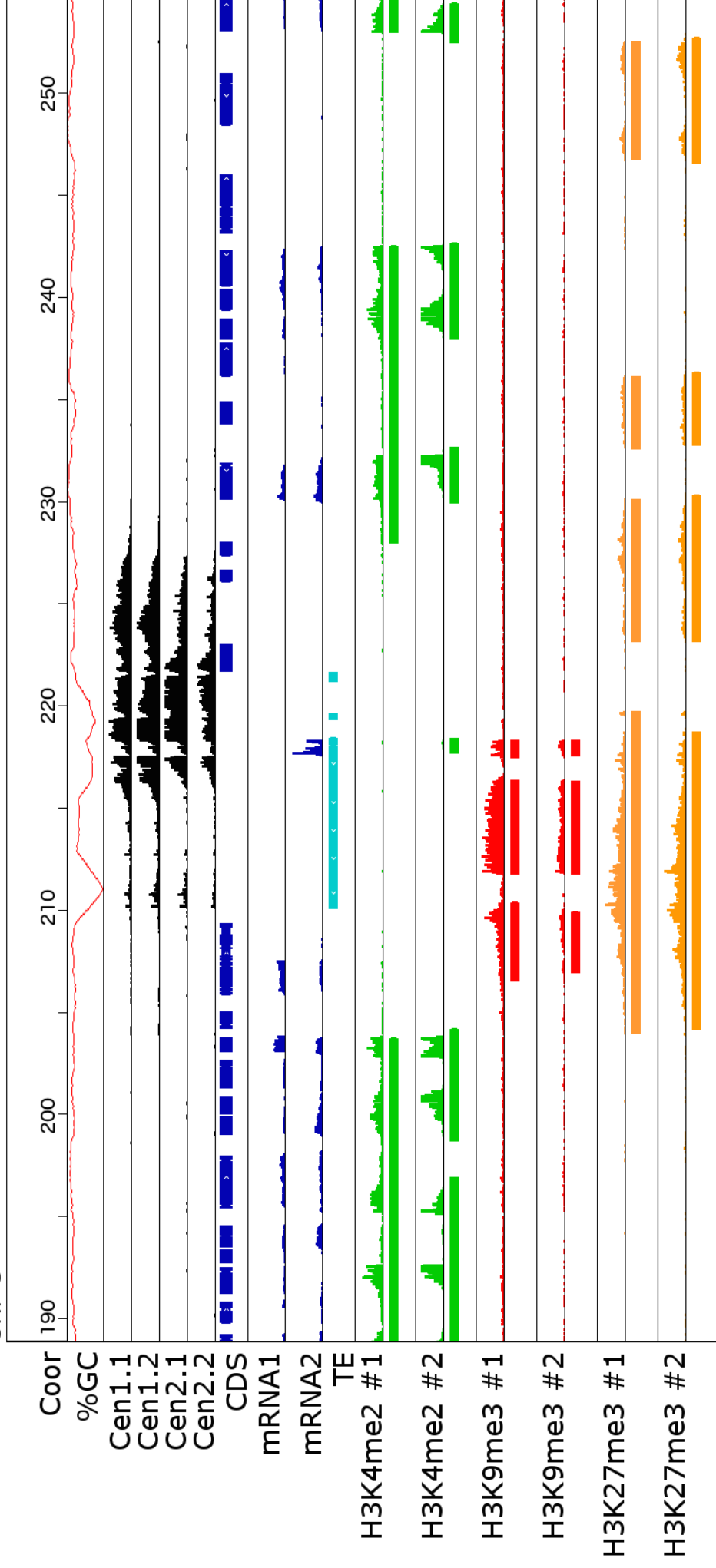

Chr 9

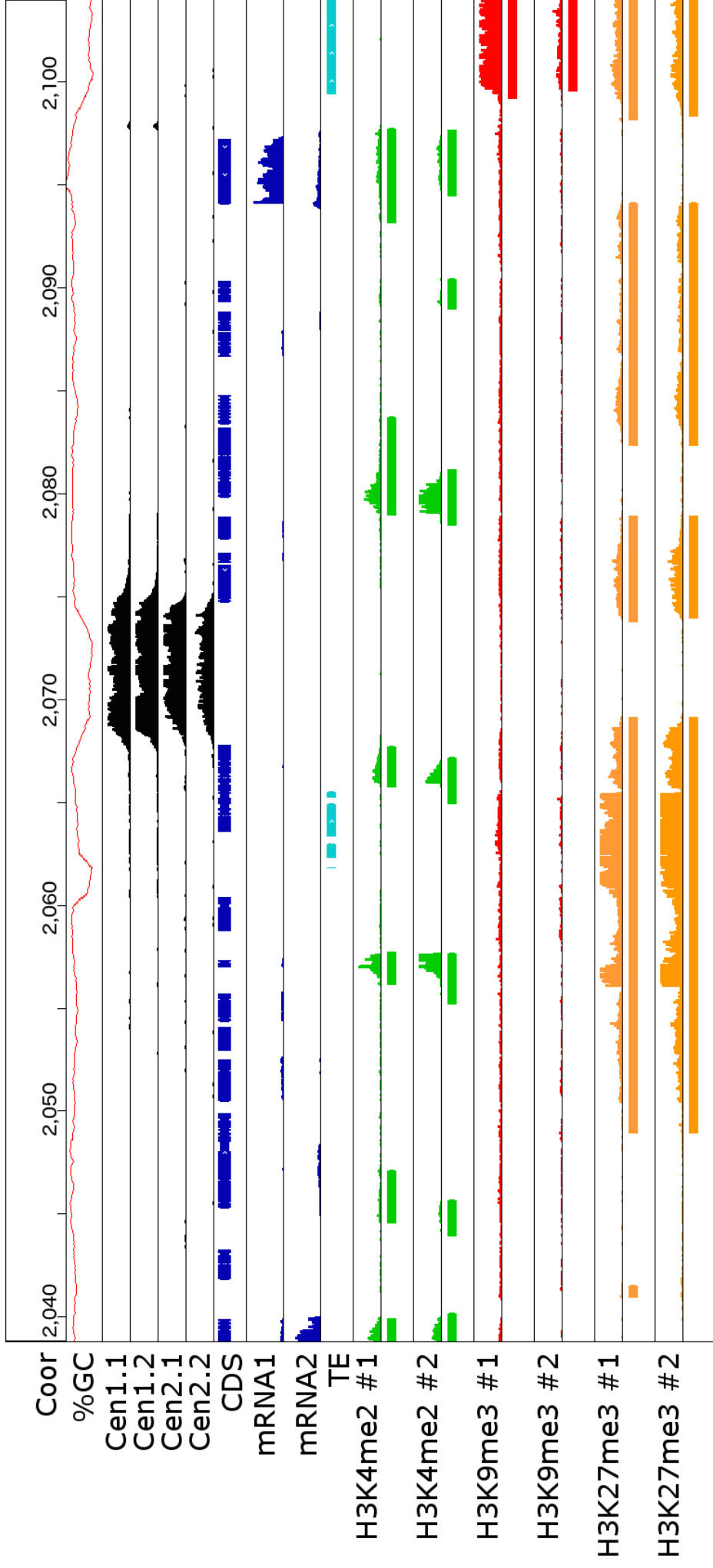

Chr 10

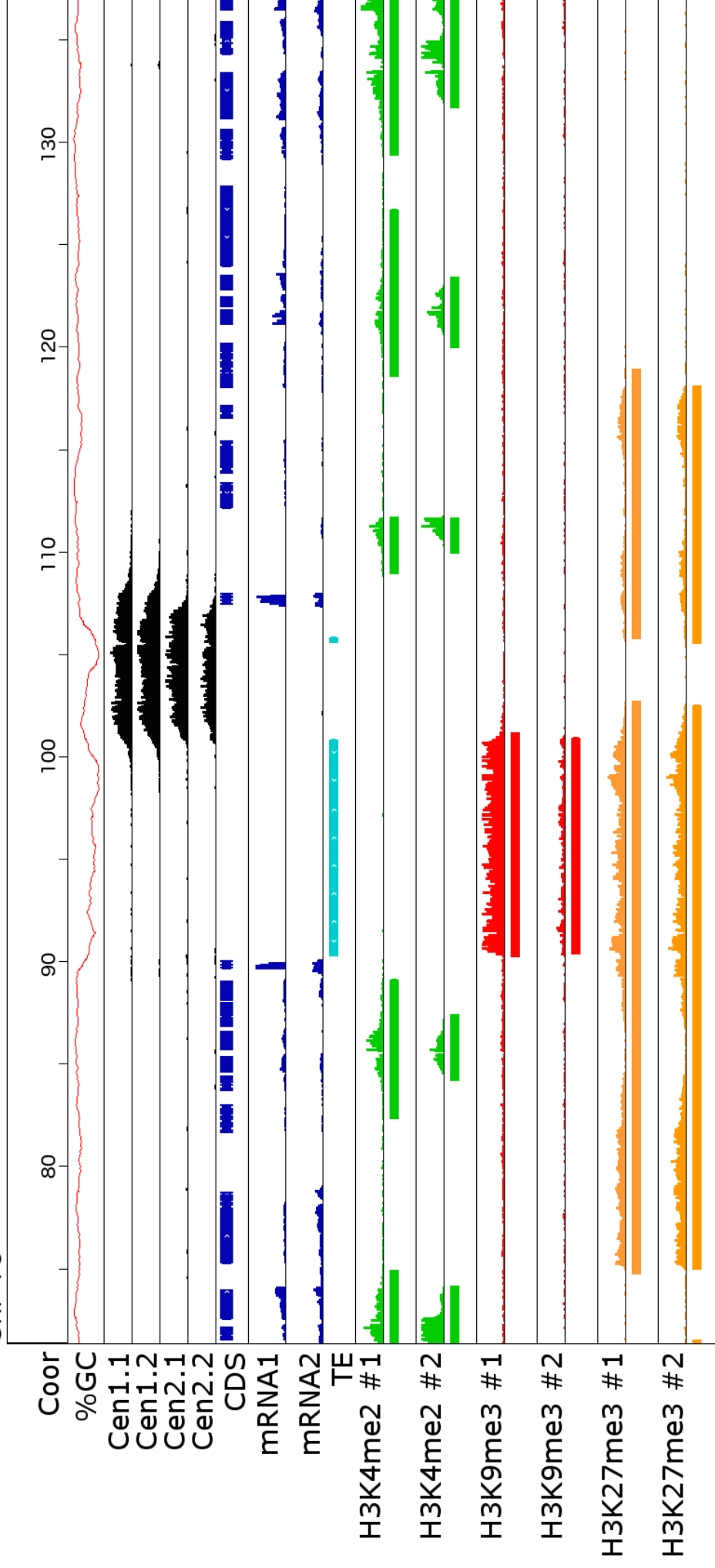

Chr 11

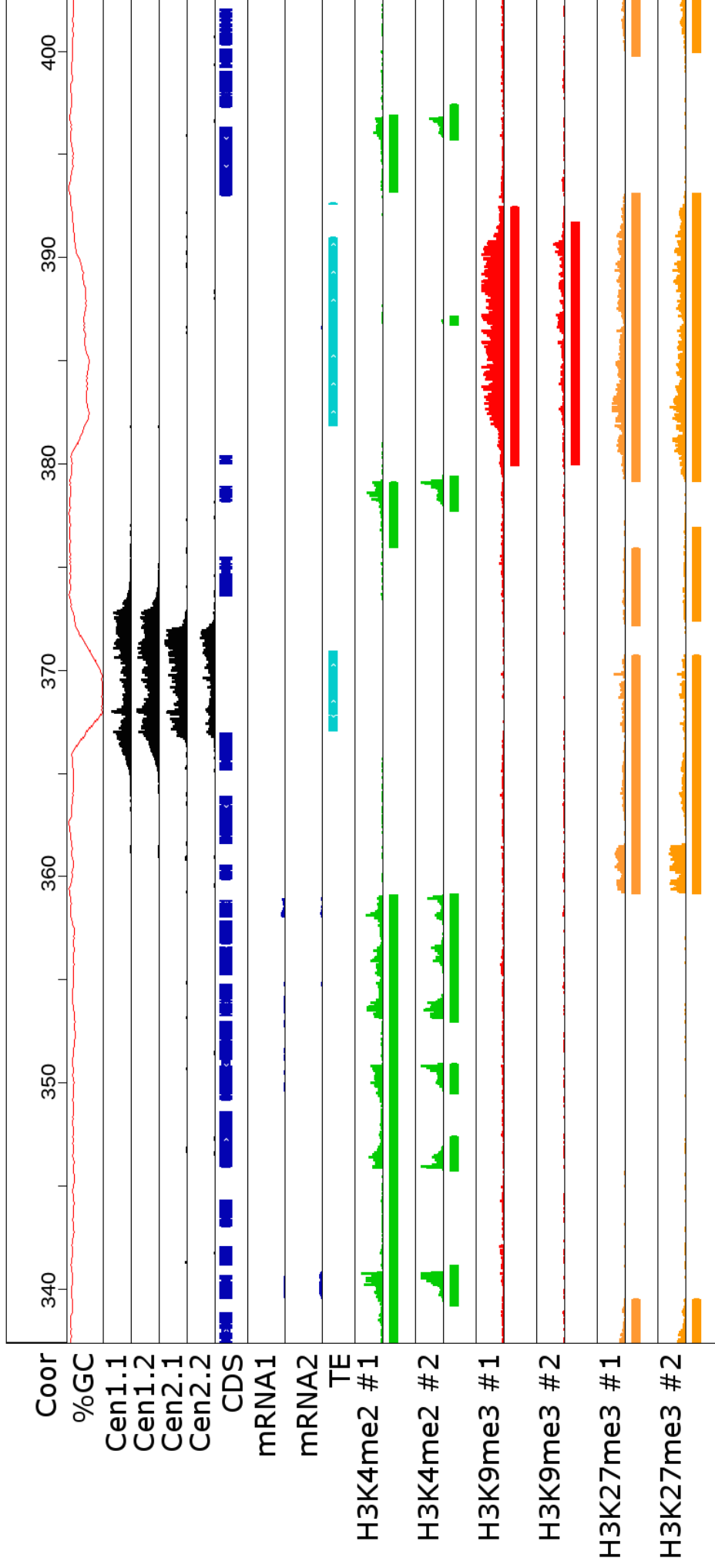

Chr 12

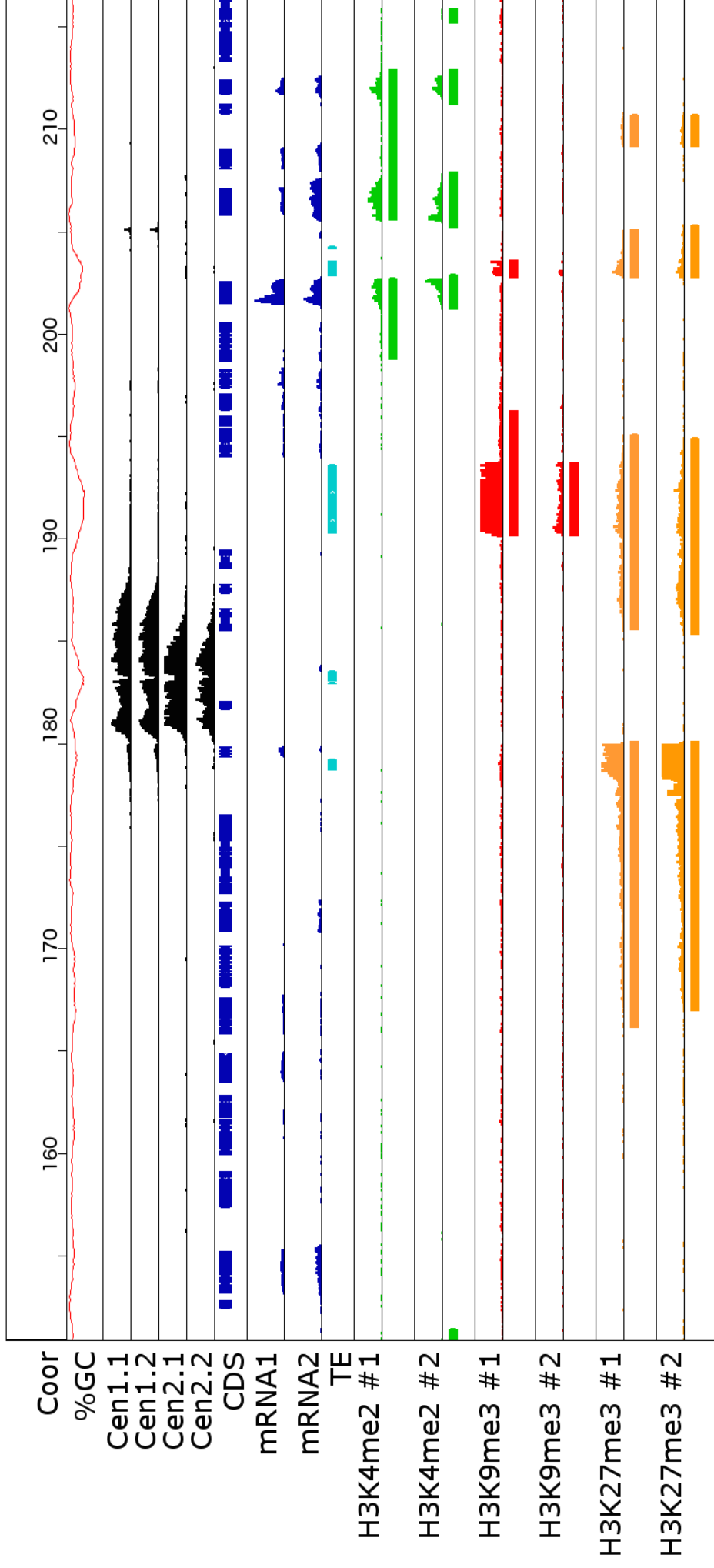

# Chr 13

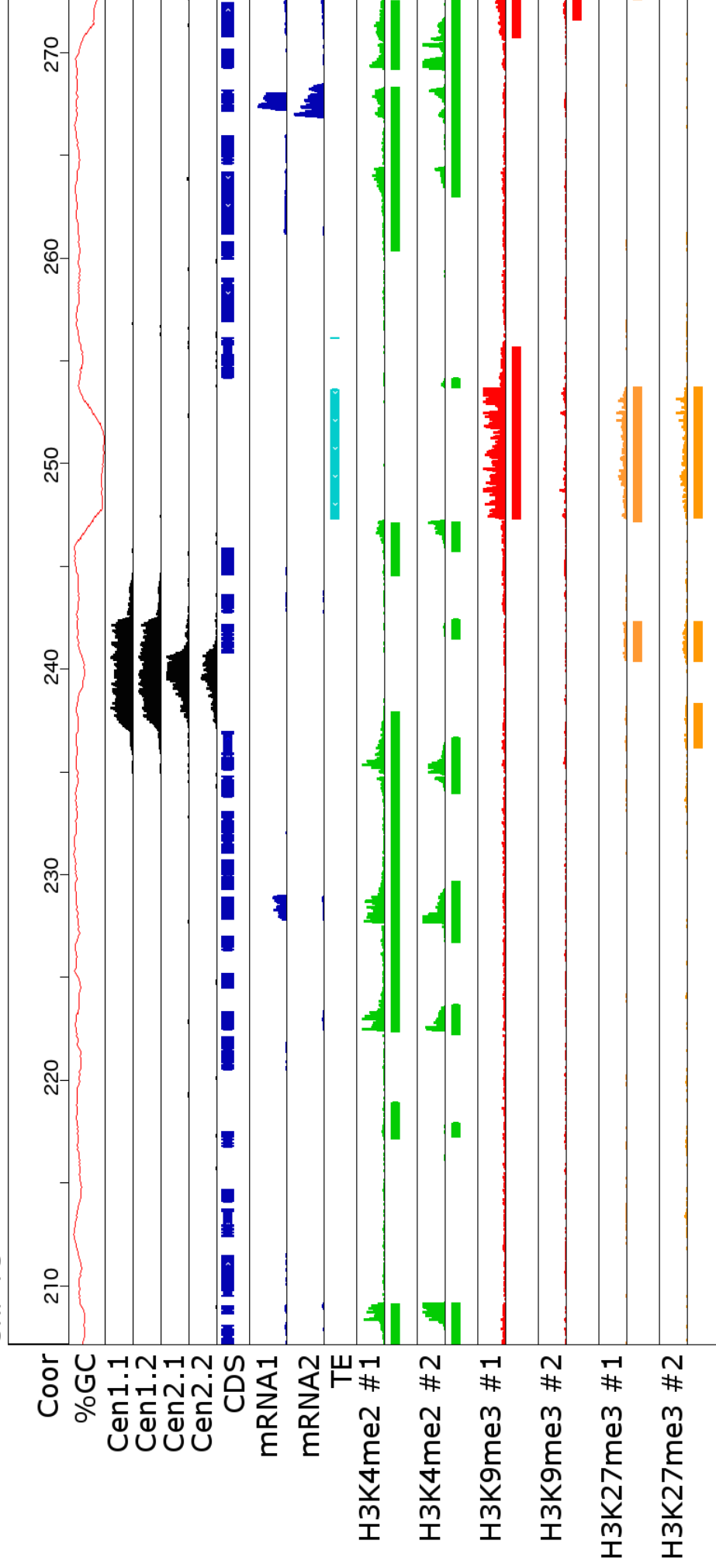

Chr 14

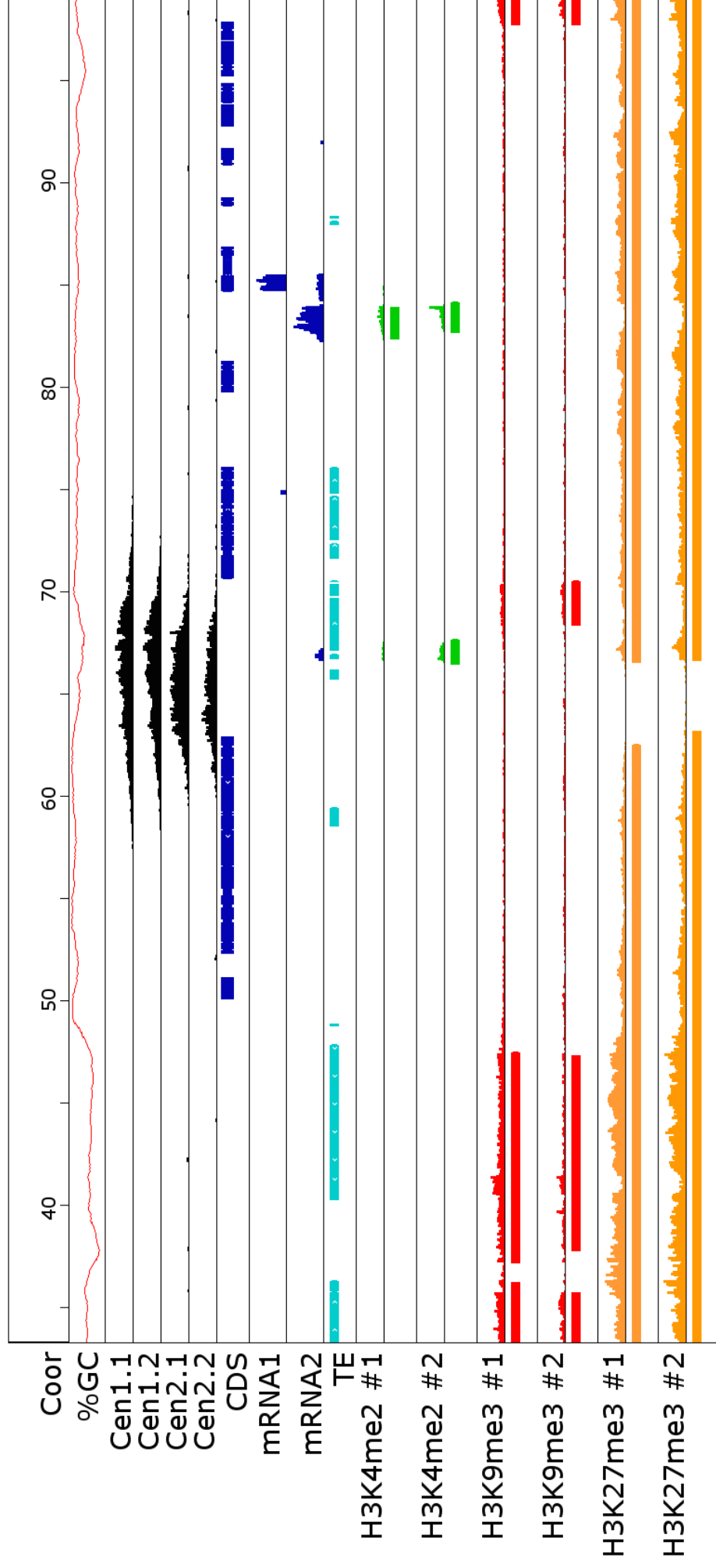

Chr 15

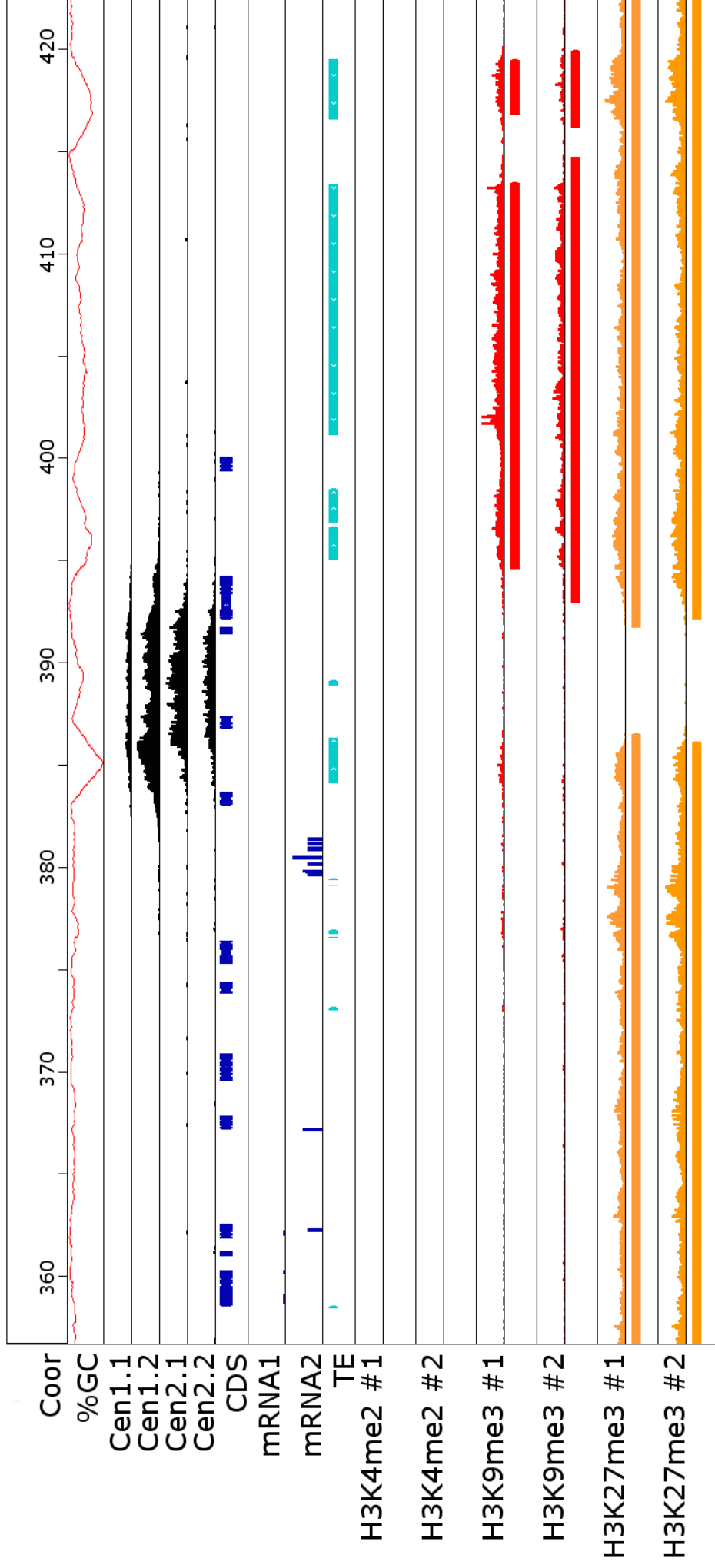

# Chr 16

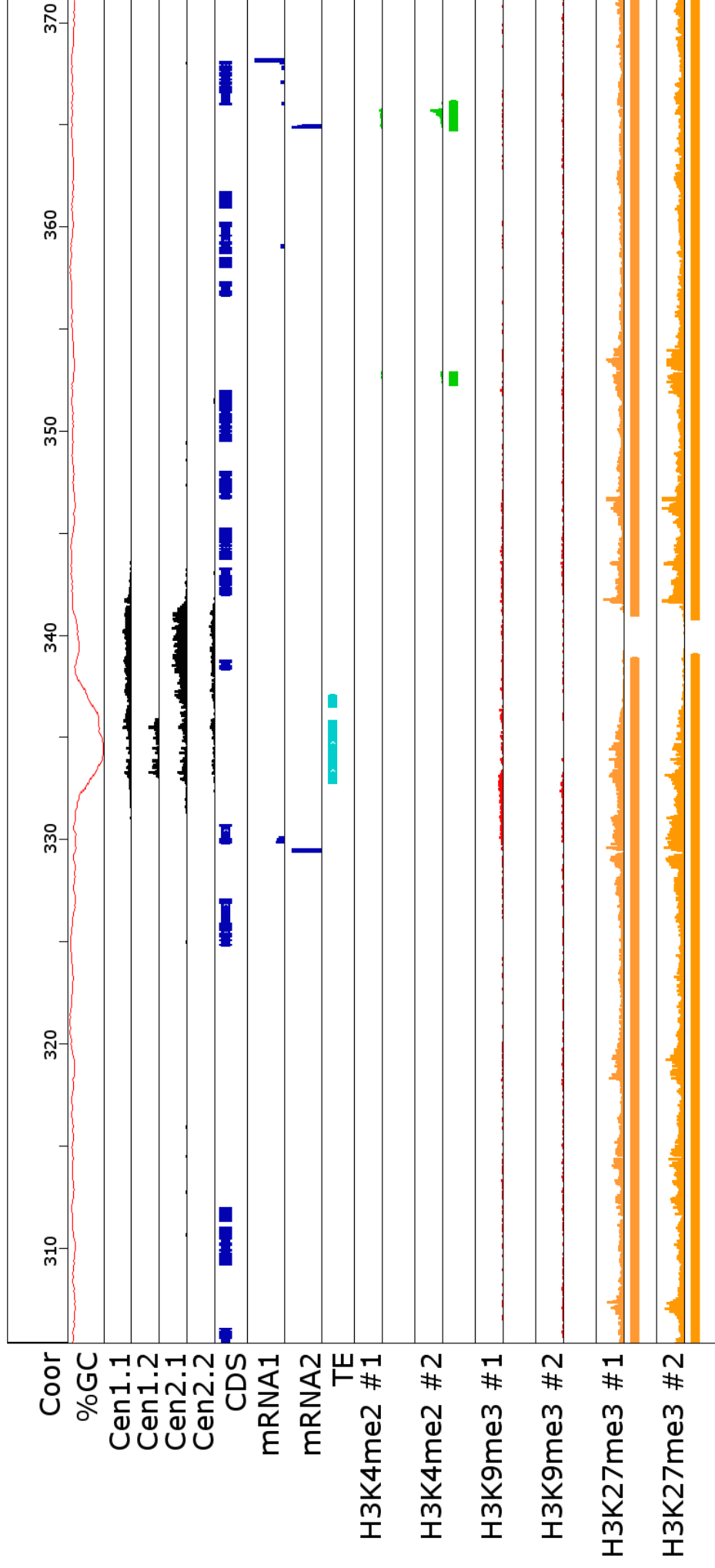

Chr 17

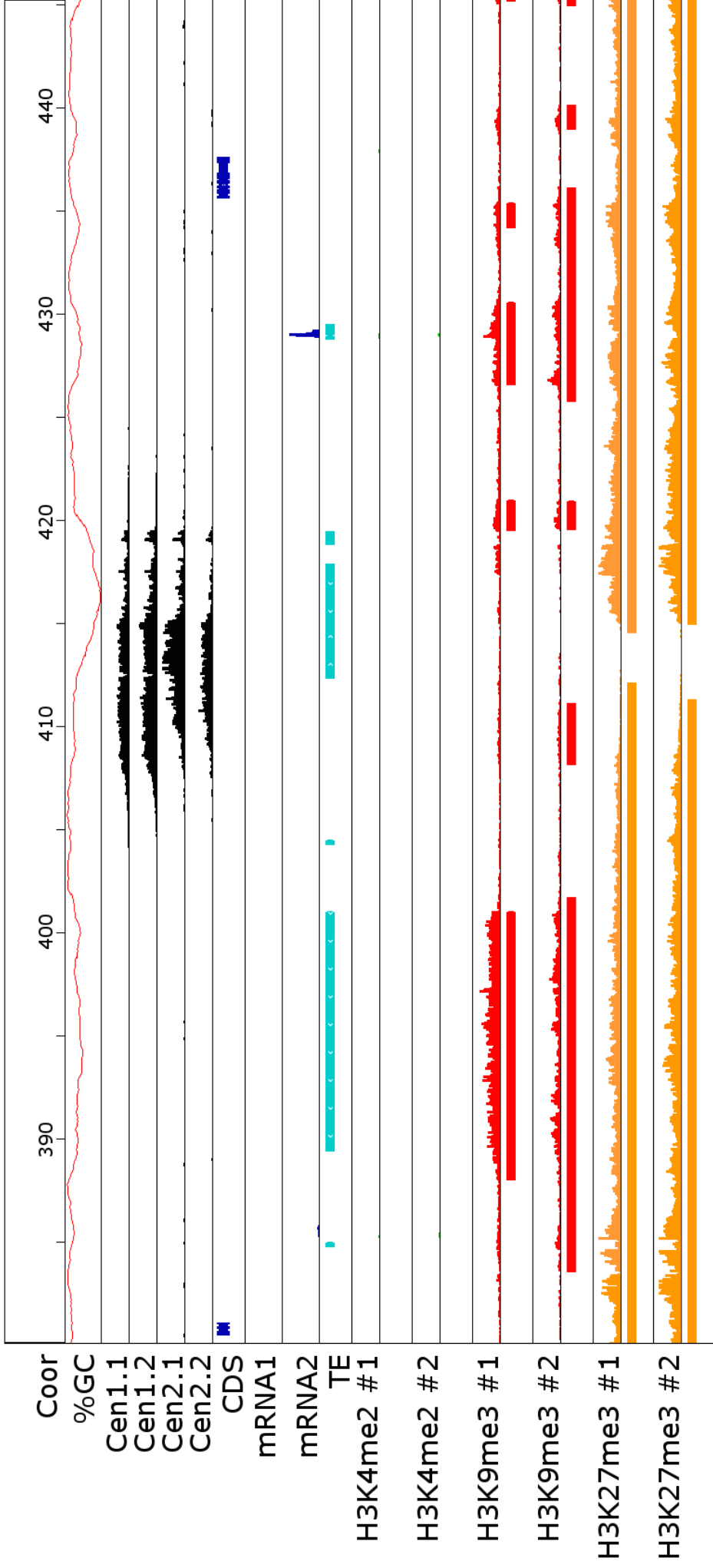

Chr 18

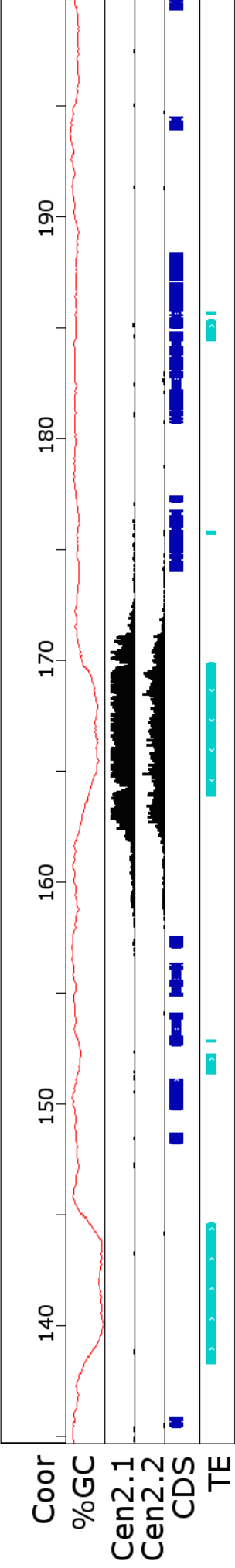

Chr 19

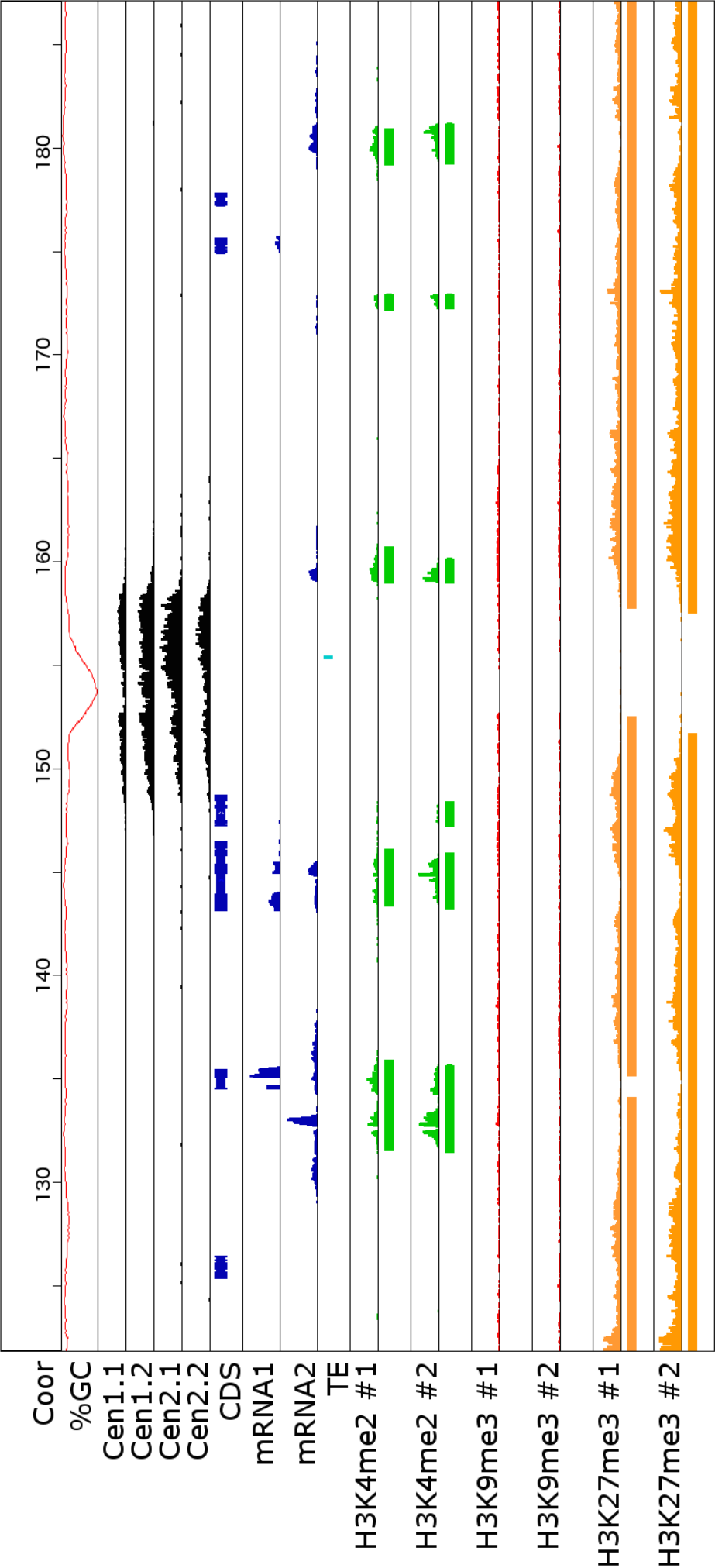

Chr 20

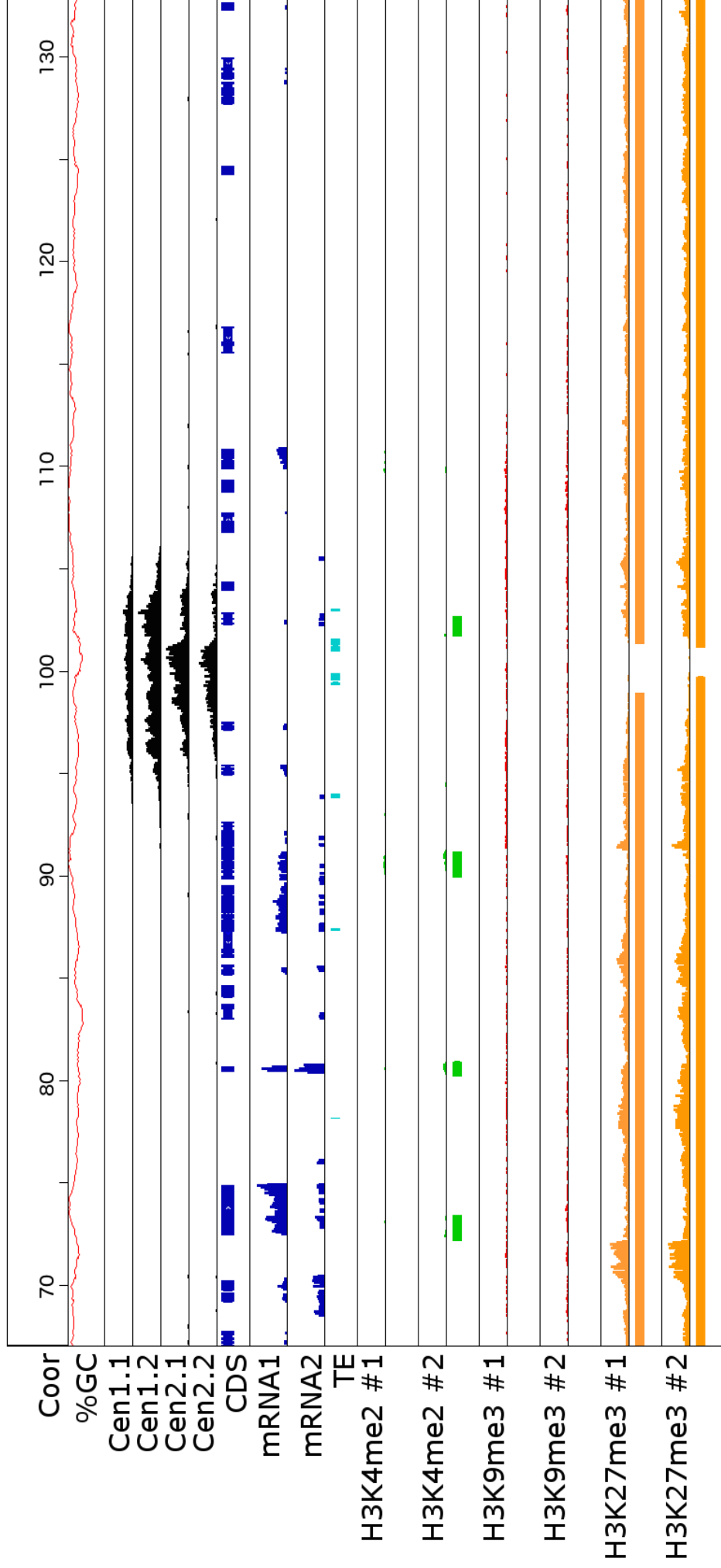

Chr 21

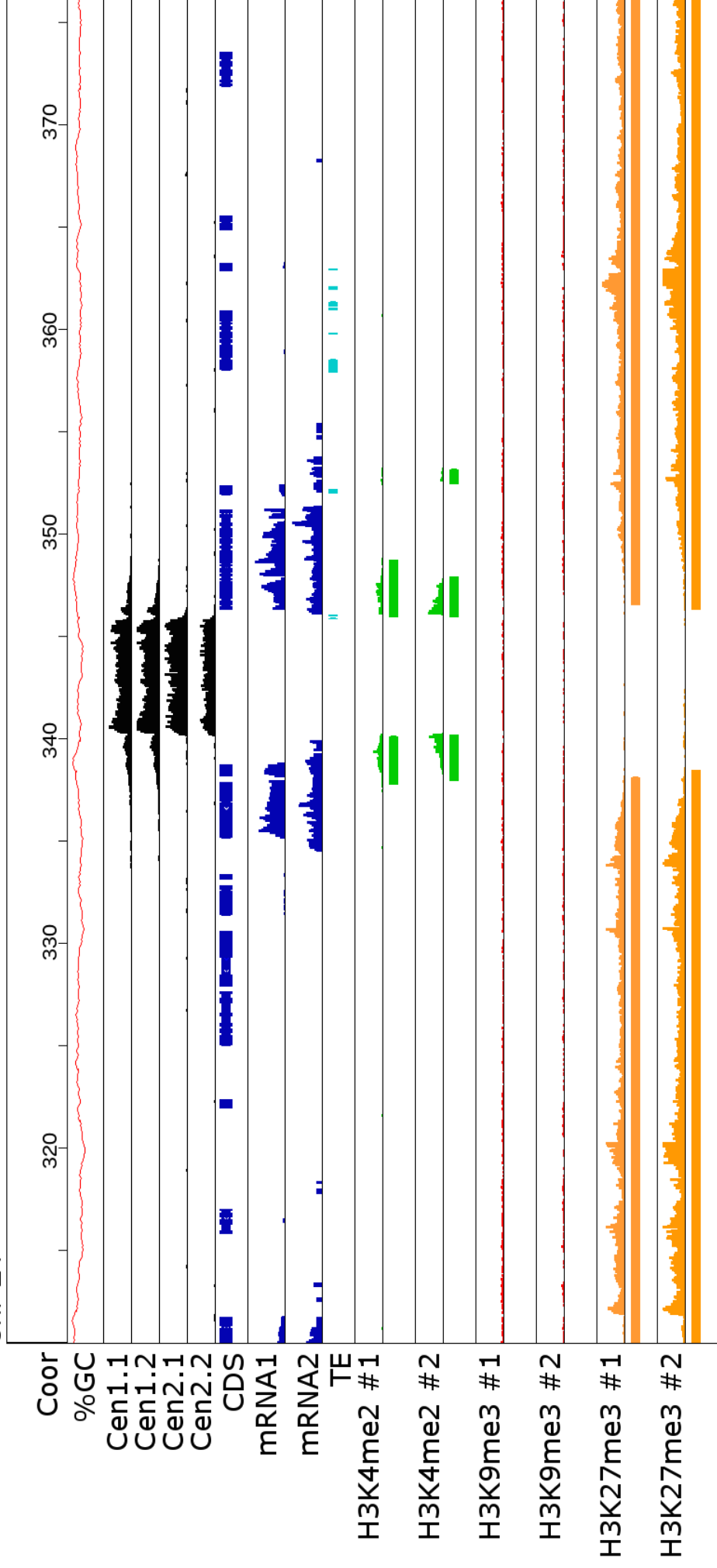

Supplement: Supplementary file 4 — 10.1186/s13072-015-0033-5 Centromere view with centromeric position and histone modifications for all chromosomes. Centromeric regions from Zt121-1 (GFP–CenH3 in IPO323ΔChr18) are shown. The ruler indicates the length of the chromosomes. For each chromosome the GC-content (%GC, red), centromeric position (Cen, black), gene density (CDS, blue), and TE content are shown (TE, marine). Enrichment with H3K4me2 (green), H3K9me3 (red) and H3K27me3 (orange) is shown. [file 13072_2015_33_MOESM4_ESM.pdf]

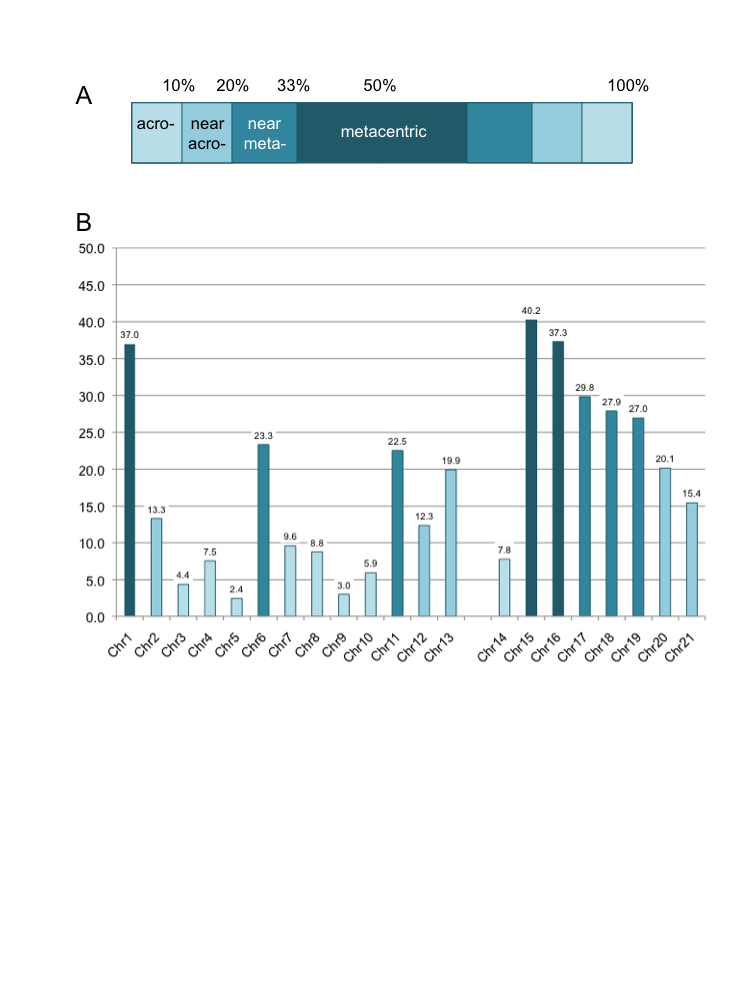

Supplement: Supplementary file 6 — 10.1186/s13072-015-0033-5 Core chromosomes of Z. tritici are mostly acrocentric or near-acrocentric while accessory chromosomes are mostly metacentric. A. Diagram outlining the definitions of metacentric and acrocentric used here. Metacentric is the middle third of the chromosomes (falling between 33 and 50 % of chromosome length counted from either telomere). Near-metacentric is chromosomes from 20 to < 33 %, near-acrocentric is chromosomes from 10 to < 20 %, and acrocentric is chromosomes from > 0 to < 10 % of chromosome length counted from either telomere. B. Core chromosomes (Chr 1 to 13) are mostly acro- or near-acrocentric, while accessory Chr 14 to 21 are mostly metacentric. Green shading indicates relative position of centromeric region on chromosomes as shown in A. The x-axis shows the chromosome numbers, the y-axis shows the relative position of the centromere on each chromosome based on the diagram in A. [file 13072_2015_33_MOESM6_ESM.tif]

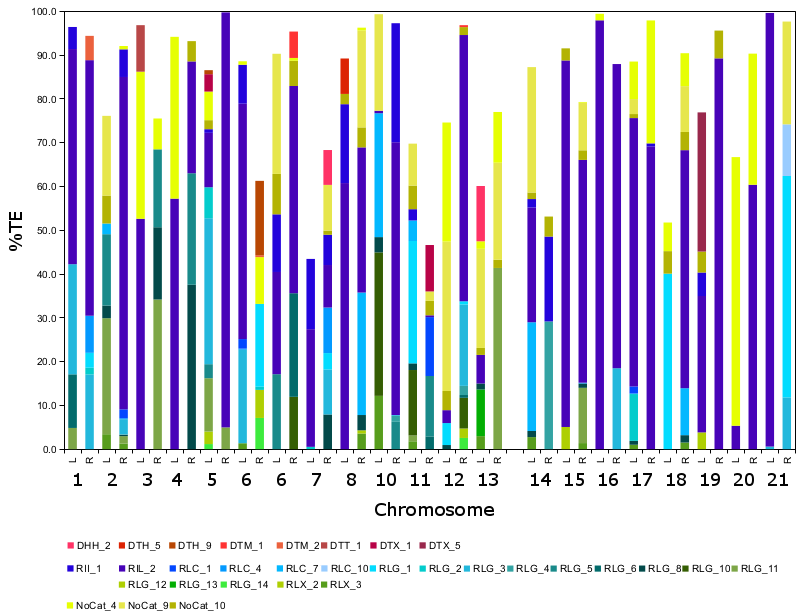

Supplement: Supplementary file 12 — 10.1186/s13072-015-0033-5 Subtelomeric regions of Z. tritici core (Chr 1 to 13) and accessory (Chr 14 to 21) chromosomes contain the same families of repetitive elements and TEs. Repeat families are labeled as described previously [12]. RIL 2 repeats make up the majority of all subtelomeric elements on both chromosome types (see also Table S5). [file 13072_2015_33_MOESM12_ESM.tif]
